# Structural basis of signal peptide recognition by the signal peptidase complex

**DOI:** 10.1038/s41467-026-73423-3

**Published:** 2026-05-22

**Authors:** A. Manuel Liaci, Dimitrios Vismpas, Lisbeth R. Kjølbye, Ioannis Skalidis, Gilberto P. Pereira, Adrian Fujiet Koh, Mariska Gröllers-Mulderij, Abhay Kotecha, Paulo C. T. Souza, Friedrich Förster

**Affiliations:** 1 Structural Biochemistry, Bijvoet Centre for Biomolecular Research, Utrecht University, Utrecht, The Netherlands; 2 Molecular Microbiology and Structural Biochemistry, CNRS UMR 5086 and Université Claude Bernard Lyon 1, Lyon, France; 3 Laboratoire de Biologie et Modélisation de la Cellule, CNRS, UMR 5239, Inserm, U1293, Université Claude Bernard Lyon 1, Ecole Normale Supérieure de Lyon, Lyon, France; 4 Centre Blaise Pascal de Simulation et de Modélisation Numérique, Ecole Normale Supérieure de Lyon, Lyon, France; 5 Thermo Fisher Scientific, Eindhoven, The Netherlands; 6 Present Address: Zymvol Biomodeling S. L., Barcelona, Spain

**Keywords:** Cryoelectron microscopy, Proteases, Enzyme mechanisms, Peptides

## Abstract

The signal peptidase complex (SPC) is responsible for cleaving signal peptides (SPs) from approximately 10% of the human proteome. SPs are characterized by a tripartite structure, consisting of an N-terminal n-region, a central helical h-region and a C-terminal c-region, each defined by rather general chemical properties rather than strict sequence conservation. Despite their sequence diversity, SPC recognizes and processes SPs with exquisite specificity. Here, we present a 2.6 Å cryo-EM map of the human SPC-A, one of two SPC paralogs, bound to a model SP. The c-region binds to a hydrophobic binding groove near the active site, a narrow gate marks the transition from c- to h-region, and the h-region localizes in a transmembrane (TM) window. Substrate engagement stabilizes N- and C-terminal helices of Sec11A, which frame the SP and are unresolved in the apo structure. Molecular dynamics (MD) simulations confirm a stable hydrogen-bonding network at the c-region and indicate dynamic interactions within a thinned lipid environment at the TM window. AlphaFold modeling supports this binding mode across physiological SPs. Collectively, our structural and computational analyses explain how the SPC achieves its specificity by combining the selectivity of the luminal binding groove and of the transmembrane window.

## Abstract (web summary)

Signal peptides are highly variable, yet the signal peptidase complex processes them with remarkable specificity. Here, authors combine cryo-EM, molecular dynamics simulations and modeling to reveal a unified binding mechanism.

## Introduction

The secretory pathway transports more than a fourth of all human proteins to the exterior of the cell, to lipid membranes or to the lumen of various organelles. Many secretory pathway proteins exhibit N-terminal, cleavable signal peptides (SPs), which are used to target the freshly-synthesized proteins to the endoplasmic reticulum (ER) membrane. The signal peptidase complex (SPC) is an essential serine protease in the ER membrane that cleaves SPs from the successfully targeted nascent chains^1,2^. It has a wide range ( > 3000) of different preprotein substrates, from which it removes highly variable SPs with exquisite specificity, leaving transmembrane helices unprocessed. In addition to its function in the biogenesis of SP-containing proteins, the SPC can also process mature, misfolded membrane proteins to initiate their degradation via ER-associated protein degradation^3–5^, activate signaling pathways^6^, and cleave polyproteins of many viruses, including flavi-, retro-, and coronaviruses^7,8^.

Given the high specificity of SP cleavage, it is surprising that different SP sequences exhibit low sequence identity. Even SP lengths vary substantially, with most SPs being in the range of ~15–30 amino acids (AAs). Instead of defined sequence motifs, rather generic structural themes define SPs. They share a tripartite structure, consisting of (i) an often positively charged cytosolic N-terminal n-region of varying length, (ii) a short ( < 18 AA, average 10–12 AA) α-helical hydrophobic h-region, and (iii) a polar c-region, which is 5–7 AA on average, as estimated by the prediction tool SignalP^9^. Signal sequences exhibit the lowest amino acid variation at positions P1 and P3 upstream of the cleavage site, with a strong preference for short, nonpolar amino acids such as alanine^1,10,11^. Another sequence characteristic is the overrepresentation of proline residues in positions P4-P6^12–14^. For the lysozyme precursor protein, the respective proline has been shown to be essential for processing.

The recently determined medium-resolution cryo-electron microscopy (EM) structures of the two human SPC paralogs SPA-A and SPC-C provide a framework for a mechanistic understanding of SP cleavage and its regulation^15^. In the largely identical paralog structures, the catalytic SPC subunit (paralog Sec11A or Sec11C, respectively) is joined by SPCS1 (also known as SPC12), SPCS2 (SPC25), and SPCS3 (SPC22/23). The transmembrane (TM) domains of the SPC collectively form a striking window within the lipid bilayer, where the lipid-detergent micelle is locally thinned to ~23 Å compared to the ~40 Å in the exterior. Coarse-grained molecular dynamics (MD) simulations indicate deformation of the lipid bilayer inside of the window that is affected by deletion and mutation of SPC subunit SPCS2^16^. If the c-region of SPs binds to Sec11 similarly as mimicking inhibitors bind to related bacterial signal peptidases^17^ the h-region could be accommodated by this window. Thus, we hypothesized a binding mode of the SP, where the shape complementarity for residues P1 and P3 at the c-region binding pocket and membrane thinning provide the required selectivity. The latter acts as a “molecular ruler“ that measures h-region length and restricts access to h-regions under 18 amino acids, as determined by systematic assays^16,18^.

In this work, we prove the hypothesized SP binding and unravel the molecular interactions enabling SPC specificity based on high-resolution cryo-EM structures of the apo SPC-A and SPC-A bound to a model SP. Molecular dynamics (MD) simulations complement the static structures, indicating robust binding at the c-region and more dynamic interactions within the TM window. Molecular modeling allow extrapolation of these findings to human SPs. Together, our analyses show that SPC specificity arises from the combined selectivity of the luminal binding pocket and the TM window.

## Results and discussion

### High-resolution structure of apo SPC-A reveals catalytic triad

**Fig. 1:**
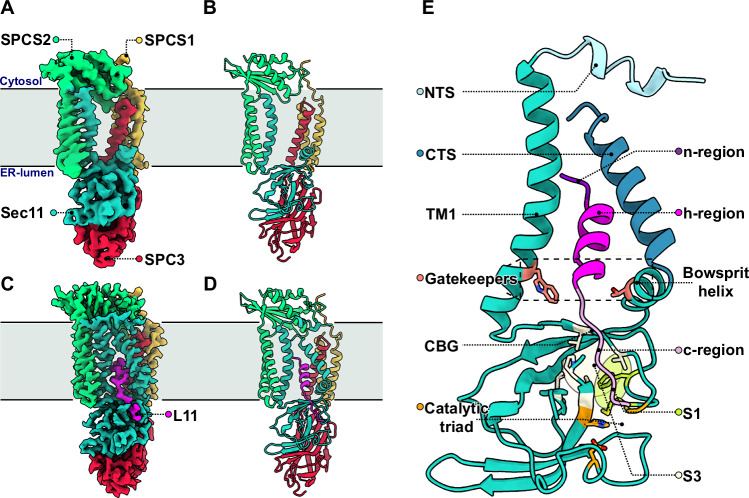
Molecular architecture of apo and SP-engaged SPC. **A** EM-map of the apo-SPC-A (Sec11A: teal, SPCS1: yellow, SPCS2: green, SPCS3: crimson). **B** Atomic model of apo-SPC-A built into the EM-map. **C** EM-map of SP^L11^-SPC-A^S56A^ complex (SP^L11^: magenta). **D** Atomic model of SP^L11^-SPC-A^S56A^ built into the EM-map. **E** Schematic overview of the Sec11A-SP^L11^ interface. NTS N-terminal short, CTS C-terminal short, TM1 transmembrane helix 1, CBG C-region binding groove.

We first aimed to obtain an atomic model of the apo SPC-A exceeding the accuracy of our previous model based on a 4.9 Å resolution cryo-EM density. To this end, we acquired a large dataset on a 300 kV transmission electron microscope (TEM), which enabled us to improve the resolution of apo SPC-A density to an overall resolution of 4.2 Å (Fig. 1A, Supplementary Fig. 1). The apo structure confirms the general architecture (Fig. 1B, Supplementary Fig. 2A, B), and the 3.5 Å local resolution near the active site allows for reliable side chain assignment of the catalytic Ser-His-Asp triad (Supplementary Fig. 2C). The improved resolution allows for a more reliable model building compared to the previous structure, as indicated by substantially higher Q-scores (Supplementary Fig. 2D). Notably, this resulted in more accurate tracing of the loop containing the catalytic Asp residue and loops in the cytosolic face of SPC-A.

### SP-based inhibitor and SPC-A form a well-ordered complex

We aimed to stabilize the SPC-A in a complex with a canonical SP to structurally investigate how SPs may bind to the SPC. Previous systematic biophysical studies of SP cleavage specificity suggested that an h-region comprising 11 leucines is approximately the median length that results in efficient cleavage by the SPC^18,19^. Analogous to this work, we designed an SP (SP^L11^) with 11 leucines framed by a positively charged n-region, a valine (^P6^Val), and a 5-residue c-region, and validated that it functions as a bona fide SP (Supplementary Fig. 4A). Two measures were introduced to stabilize the complex and prevent cleavage by the cellular background: (i) we mutated Sec11A’s catalytic serine to alanine (SPC-A^S56A^) to prevent substrate cleavage and release, and (ii) we added a proline at the position P1’ of the SP, inspired by a previously reported mutated preproinsulin SP that prevented cleavage of the substrate by the SPC and allowed co-immunoprecipitation with the SPC^20^. Co-expression of this designed SP^L11^ with SPC-A^S56A^ in HEK293 cells indeed allowed co-purifying the enzyme with the peptide (Supplementary Fig. 4B).

### CTS and NTS helices are ordered in the SP-SPC complex

Using a cryo-TEM equipped with latest generation energy filter and detector we obtained a cryo-EM density of the SPC-A^S56A^-SP^L11^ complex in DDM/CHS with an overall resolution of 2.6 Å, displaying clear side-chain densities in most parts of the complex (Fig. 1C, D, Supplementary Fig. 3, Supplementary Fig. 4C). The notably higher resolution of this complex compared with the apo SPC suggests that the substrate-bound complex is structurally more homogeneous. In addition to protein density, the map also displays various ordered lipid or detergent molecules in the proximity of the TM window, which we did not analyze further (Supplementary Fig. 4D).

In addition to the SP^L11^, the engaged SPC-A^S56A^ also displays well-ordered short N- and C-terminal helices of Sec11A (NTS and CTS helix, respectively, (Fig. 1E), which are not resolved in the apo structure. Both helices primarily form contacts with themselves and the SP^L11^. The absence of these helices in the apo map, which is irrespective of the detergent being used (DDM/CHS vs. PMAL-C8, Supplementary Fig. 2A), suggests that both the NTS and CTS helices position flexibly in the absence of substrate, and latch into place upon SP recruitment. The ~20-residue CTS helix can be traced traversing the membrane, in line with our earlier hypothesis that the CTS helix is a TM segment^15^. Remarkably, the amphipathic N-terminal 3-turn NTS helix forms close, mainly hydrophobic contacts with the CTS helix. While the NTS helix does not bind to the SP directly, it appears to stabilize the positioning of the CTS by forming a “lid”.

### The h-region of SP^L11^ associates with Sec11A CTS and TM1

**Fig. 2:**
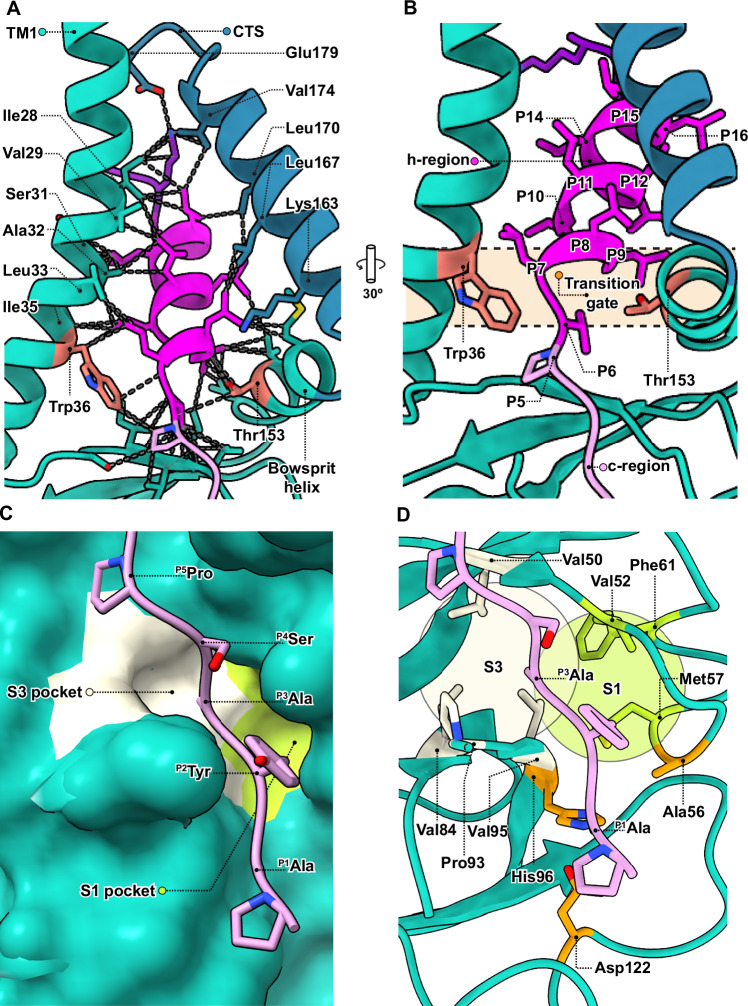
SP^L11^ accommodation in the SPC catalytic subunit Sec11A. **A**, The helical gear formed by TM1, SP^L11^ and CTS. Van der Waals interactions are indicated in dotted dark gray lines (overlap ≥−0.6 Å). **B**, Gatekeeper amino acids (salmon) define the SP^L11^ transition point from h- to c-region. For depiction, the transition gate framed by the gatekeepers is defined between the dotted lines (pale orange). Visible SP-residues are annotated. **C, D**, Surface (**C**) and atomic (**D**) representations of the CBG. S3 and S1 pockets are highlighted (beige and lime-green, respectively). The His-Asp-Ala mutated catalytic triad is annotated in orange.

While the n-region of SP^L11^ does not show interactions with the SPC-A and is only partially resolved, most other parts are highly ordered in the SPC-A^S56A^-SP^L11^ structure, indicating a tight association in a single conformation. Direct interactions of the h- and c-region are exclusively formed with the catalytic subunit Sec11A. The Sec11A transmembrane helix 1 (TM1) and the CTS helix form a “helical gear” with hydrophobic spokes interacting with some of the h-region residues of SP^L11^ (P7-8, P10-P12 and P14-15) (Fig. 2A). We have previously shown that the CTS helix is necessary for the catalytic function of the SPC^15^, which underscores the functional importance of this interaction.

### Gatekeepers localize at the transition point between h- and c-region

Upon transitioning from the TM space to the lumen, and concomitantly from the h-region to the c-region, the SP^L11^ unwinds into a β-strand conformation (Supplementary Fig. 5A). This structural change occurs in the line defined by a narrow gate, laterally formed by the “gatekeeper” amino acids Trp36 and Thr153 residing in TM1 and the bowsprit helix, respectively (Fig. 2B), which are evolutionary highly conserved (Supplementary Fig. 5B). The ^P6^Val of SP^L11^ is wedged between the bulky TM1-Trp36 and the side chain of Thr153, which sterically block further extension of the helix to the lumen. The secondary structure transition is further reinforced by the presence of ^P5^Pro. Consistent with the helix breaking properties, Pro residues are statistically enriched at the P4-P6 position of SPs^12–14^. The precise role of the gatekeeper residues remains to be addressed in future mechanistic studies.

### C-region binding groove (CBG) hosts the SP c-region

A hydrophobic ridge on the luminal Sec11A domain, which we termed the c-region binding groove (CBG), connects the TM window to the catalytic triad. The backbone atoms of P4-P2 of the β-strand-like c-region are involved in an extensive backbone interaction network with Sec11A (Supplementary Fig. 5C). The P4 and P2 side chains project to the membrane, explaining the moderate residue specificity at these sites. As in the bacterial SPase I and LexA structures^17,21^, the CBG groove contains two shallow, hydrophobic pockets, canonically called S3 and S1 (Fig. 2C, Supplementary Fig. 5D) because they engage the P3 and P1 residues, respectively. Residues Val50, Val52, Val84, Pro93, and Val95 form S3, which harbors the small and nonpolar side chain of ^P3^Ala. Pocket S1 comprises residues Met57, Phe61, Val52, and Val95 with the latter two shared with S3. While S3 is occupied by ^P3^Ala (Fig. 2D), we observe only a weak EM density inside the S1 pocket and in proximity to the catalytic triad, which can best be explained by a fractional ( ~ 30%) occupancy of ^P1^Ala (Supplementary Fig. 6), although we cannot fully exclude alternative explanations. The main fraction of ^P1^Ala projects away from the S1 pocket toward the solvent, presumably due to the stereochemical restrictions imposed by the inhibitory proline in position P1’. Thus, the low occupancy of the S1 pocket may explain the reported inhibitory effect of the proline at P1’^20^, in analogy to findings for the bacterial type I SPase SpsB from *S.aureus*^22^.

### Lipid thinning and dynamics of the SPC-L11 interaction

**Fig. 3:**
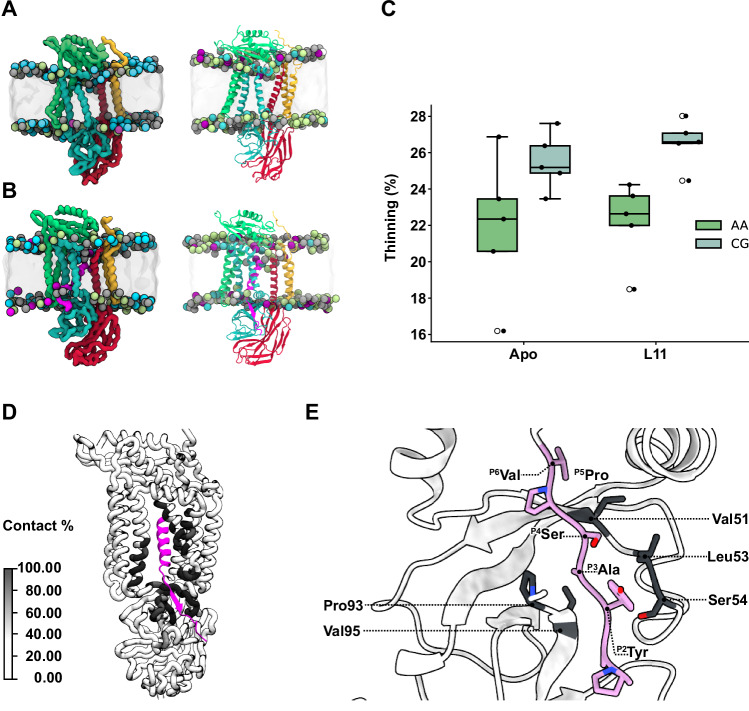
Molecular dynamics simulations of the SPC-SP^L11^ complex. **A**, Snapshots of apo-SPC at a coarse-grained (CG, left) level and atomic representation (AA, right). The lipid phosphate groups are illustrated with spheres (POPC: grey, POPS: magenta, POPE: cyan blue PIP2: green). The lipid tails are shown in light grey. **B**, Snapshots of the SPC-A^S56A^-SP^L11^ complex as in A. **C**, Comparison of the thinning percentage of the lipid membrane in the lateral window at CG and AA resolution, respectively for both the Apo and L11 complex. The boxplots show the interquartile range (IQR), with center lines indicating the median and whiskers spanning the full data range. Hollow circles mark outliers beyond the whiskers. Filled circles are the independent simulation samples (*n* = 5). Summary statistics are reported as mean ± SD, with full ranges as follows: AA Apo: 21.89 ± 3.93 (median 22.35, range 16.19–26.87). AA L11: 22.19 ± 2.25 (median 22.64, range 18.49–24.24). CG Apo: 25.50 ± 1.57 (median 25.19, range 23.46–27.61). CG L11: 26.53 ± 1.30 (median 26.59, range 24.46–28.02). **D**, SPC-A^S56A^-SP^L11^ contact frequencies for the bound state mapped on the Sec11A structure from AA simulations. **E**, Residues contacting SP^L11^ for over 60% of simulation time are highlighted (dark grey). Source data are provided as a Source Data 1 file and in Zenodo (DOI: 10.5281/zenodo.18299233).

To assess the dynamics of a SP prior to cleavage, we performed MD simulations (Fig. 3) both at atomistic and coarse-grained resolution. As there are no experimental restraints on the simulation, we reverted both the S56A mutation of Sec11A and the Proline residue at P1’, and we added a 7-amino-acid C-terminal stretch to better simulate a nascent preprotein (SP^L11^) (Supplementary Fig. 7A).

The initial pose of SP^L11^ within the SPC was based on an AlphaFold2-Multimer model (AF2)^23^, which includes protein segments that were not modelled in the EM structure. This model was compared and validated against our cryo-EM structure. With alignment on the backbone of Sec11A, the backbone root-mean square deviation of the Sec11A domain and the peptide is 1.9 and 2.1 Å, respectively, between the AF2 model applied in the MD simulations and the cryo-EM structure. To equilibrate the lipid membrane, the complex was embedded into an ER lipid membrane model^15^ and simulated at a coarse-grained (CG) level, restraining the protein complex, while the membrane would relax around it. After 1 µs of simulation time, the system was converted into an atomistic resolution, and simulations were re-started, simulating a period of 1 µs (Fig. 3A-B). All the simulations were repeated as five independent samples. The CG simulations were further extended for 12–20 µs for the apo and peptide bound systems, respectively, removing the restraints to allow the protein complex to change and adapt.

The inclusion of the lipid membrane allowed for quantifying the membrane thinning within the TM window. The reduction of the thickness of the lipid bilayer compared to areas distal from the SPC was found to be approximately 22% in the atomistic simulations (Fig. 3C). This trend in thinning from atomistic MD agrees with the thinning observed at CG resolution over longer timeframes, which is slightly higher at ~25%. The thinning is more pronounced at CG resolution across all simulations, likely because of increased sampling. Thus, the MD simulations strongly support the notion that the TM window promotes a thinned lipid environment for hosting SPs.

During the atomistic simulations, the SP quickly adopted a cleavage-competent state, with the c-region firmly inserting itself into the CBG. The residues lining the CBG displayed the highest contact frequencies (60% or above) (Fig. 3D, E) while the CG simulations showed less contact to the S1 and S3 pockets. The most consistent interactions were β-sheet-like hydrogen bonds contributed by residues Val51 (to ^P5^Pro and ^P3^Ala), Leu53 (to ^P3^Ala), Pro93 (to ^P2^Tyr), Val95 (to ^P2^Tyr), and Ser54 (to ^P1^Ala), which create a local hydrogen-bonding network with the backbone of the SP^L11^ c-region. Both ^P3^Ala and ^P1^Ala most frequently occupy the S3 and S1 pocket, respectively (Supplementary Fig. 7B). The main contact points in SPC-A were confirmed in another setup with a natural peptide from human Integrin alpha-IIb (Uniprot ID: P08514).

Compared with the c-region, the h-region interacts more variably with the SPC, with the tilt angle of the helix relative to the membrane normally varying from 20° to approximately 50°, with a median at 30° (Supplementary Fig. 7C, D). These results are consistent across the atomistic and CG simulations. In the h-region, the highest interaction frequency occurs for the lower TM1 residues towards the lumen, the bowsprit helix, and the CTS helix, including the two gatekeeper residues Trp36 and Thr153. The TM helix of SPCS2 displays a limited set of contacts (Fig. 3D), not appearing as a main contact player. Thus, based on our MD simulation, the β-sheet hydrogen bonding network between the backbone residues of the CBG and the SP positions P5 to P1 provides the strongest interaction with a contact percentage of 76–96% across the 5 atomistic simulation repeats. In contrast, the non-directional hydrophobic interactions provided by the TM window provide a more flexible binding environment with more transient interactions, which pivot around the gate formed by Sec11A’s gatekeeper residues.

Local membrane thinning has previously been demonstrated to enhance proteolytic activity in the rhomboid protease GlpG, which locally modulates the lipid bilayer around its intramembrane active site^24–26^. In contrast to GlpG, the SPC active site resides in the ER lumen. The lipid thinning observed within the SPC TM window in our MD simulations may influence proteolytic function over a great distance due to the long recognition motif that characterizes an SP.

### Modelling engagement of human SPs

Physiological SPs display remarkable variability both in terms of sequence and length. Our previous analysis of all experimentally verified human SPs using SignalP^27^ indicated that almost all of the length variability originates from the n-region^15^ which, according to our structure, does not interact with the SPC. Meanwhile, we observed only a moderate length variation in both the h- and c-regions around their median values (12 and 5 residues, respectively). Thus, this analysis suggested that interaction of the c- and h-regions with the SPC may be much less variable than SP length and sequence variation among native SPs might suggest. The question is how and to what extent the SPC-A^S56A^-SP^L11^ may capture a main mode of interaction for the diverse class of SPs.

Since it is impossible to sample a significant fraction of SP variation experimentally, we attempted to evaluate the potential of modeling SP engagement computationally using AlphaFold2 (AF2) multimer^23^ for physiological SPs^28^. We first assessed the predictive accuracy of AF2 using the structure of the Sec11A-SP^L11^ interaction (Supplementary Fig. 8A). While AF2 predicted most parts of the SPC-A^S56A^-SP^L11^ complex with high confidence, the pLDDT scores for Sec11A-SP^L11^ interaction were slightly higher for the binary Sec11A-SP^L11^ model. Comparison of this AF2-model with the experimentally determined counterpart reveals a strong agreement in the positioning of the h- and c-regions of the SP^L11^ peptide, while there are notable discrepancies in the n-region and the CTS/NTS helices (Supplementary Figure 8A). Importantly, the pLDDT confidence scores of the AF2 model are also high (pLDDT > 70) for the SP^L11^ residues ( ± 5) flanking the transition point, where experimental and AF2 model are almost indistinguishable (Cα-RMSD < 0.6 Å), with the notable exception of P1, where the inhibitory proline at P1’ likely prevents insertion into the S1 pocket.

**Fig. 4:**
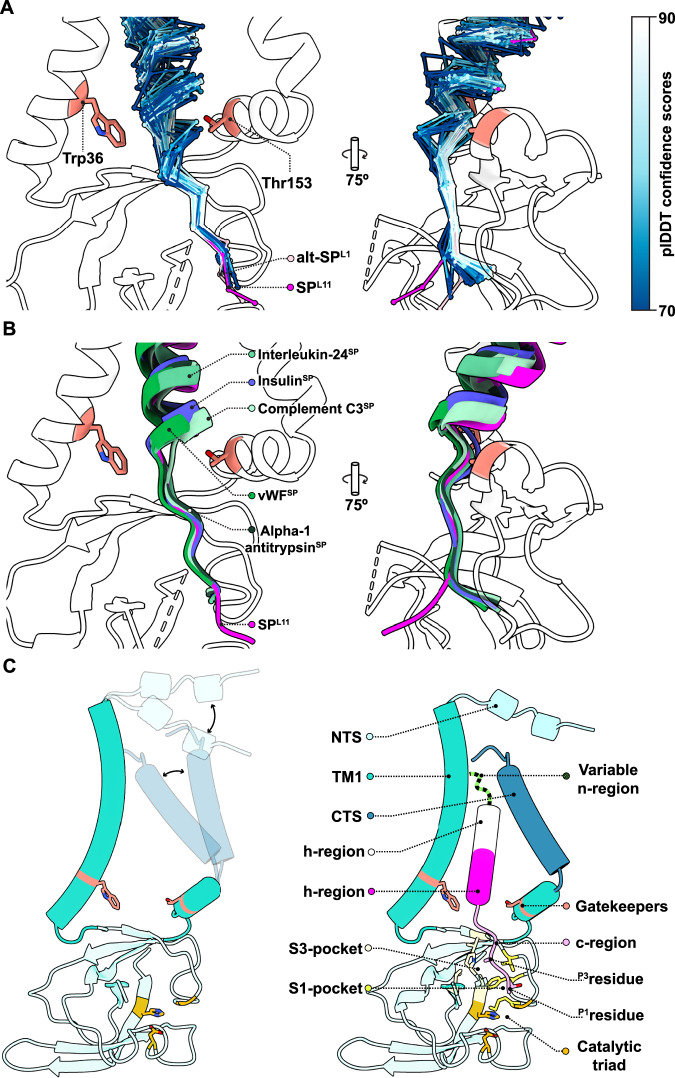
AlphaFold2 models indicate generality of SP^L11^’s mode of engagement. **A**, Transition point of AF2 predicted SP models (plDDT>70, *N* = 92) superposed on experimental SPC-A^S56A^-SP^L11^ structure (white). Source data are provided as a Source Data 2 file and in Zenodo (DOI: 10.5281/zenodo.18299233). SP^L11^: Main experimental conformation (magenta), alt- SP^L11^: lower occupancy, alternative conformation. **B**, Cartoon representation of high confidence AF3 predictions (plDDT>70) superposed on the experimental SPC-A^S56A^-SP^L11^ structure. Insulin^SP^ (P01308), von Willebrand Factor^SP^ (P04275), Interleukin-24^SP^ (Q13007), Complement C3^SP^ (P01024), and Alpha-1-antithrypsin^SP^ (P01009). **C**, Proposed general model of SP accommodation in the catalytic subunit Sec11A. Left, Sec11A prior to SP binding. Right, the proposed model of SP-accommodation; Stabilization of the h-region between TM1 (teal) and CTS (dark-blue) and seal-off by the NTS (light blue). Gatekeeper residues (salmon) define the region transition. Residues P3 and P1 engage pockets S3 (beige), S1 (yellow). Structural variability in n-region is illustrated with green-black. h-region length variability occupying the TM-window is shown with light pink.

Encouraged by this result, we co-modeled all 412 experimentally validated human SPs^15^ with Sec11A. Low-confidence models as well as those displaying reverse orientation within the CBG (AF2 failure mode I) or register-shifted c-regions with respect to the cleavage site (AF2 failure mode II) (Supplementary Fig. 8B) were excluded, retaining 92 models (Supplementary Fig. 8C). The retained Sec11A-SP models converge at the CBG and the transition gate (Fig. 4A). All high-confidence models have a 5-residue c-region and strongly resemble the experimental Sec11A-SP^L11^ structure (Fig. 4B, Supplementary Fig. 9).

Overall, the structural variation of high-confidence SP models from SP^L11^ is low at the c-region (again with the exception of P1) and increases in the h-region distal from the gate (Supplementary Fig. 10A, B), which is qualitatively similar to the variation of SP^L11^ observed over time in our MD simulations. In summary, AF2 modeling suggests that >20% of human SPs bind to the SPC similarly as in the SPC-A-SP^L11^ structure, and there is no indication that strongly differing binding modes exist.

### SPC structure in context with ER translocon

In the cellular context, SP cleavage occurs after translocation of the SP towards the ER lumen via the dynamic ER translocon complex^29^. Its core component Sec61 accomplishes translocation, which is predominantly a co-translational process in mammals. Although ex vivo assays showed that SP cleavage can be performed post-translationally^30^, this process is believed to mostly occur co-translationally in the cell^31^. Indeed, ex vivo experiments demonstrated that the SPC can cleave SPs co-translationally, most comprehensively in ref. ^32^. These studies indicated that approximately 40 amino acids are required between Sec61 exit and SPC active site for co-translational cleavage. Consistent with the notion of co-translational processing, chemical cross-linking indicated transient proximity of the actively translocating Sec61 subunit Sec61β and SPC subunit SPCS2^33^. The SPC-SP^L11^ structure indicates that proximal localization of SPCS2 to Sec61β would position the SPC close to the lateral gate of Sec61α enabling most direct insertion of the SP into the SPC membrane window. In the context of the native ER secretory translocon structure^34^, the SPC would then reside in the vicinity of the translocon-associated protein complex (TRAP), which modulates the ER lipid membrane shape^35^. Thus, lipid–protein interactions involving ER translocon and SPC may profoundly impact early biogenesis of secretory pathway proteins. Future experiments in the native ER membrane will be required to address the interplay of ER translocon and SPC and possible differences among its paralogs.

### Principles of signal peptide recognition by SPC

In this study, we reveal the association of a model SP to the SPC and explore its temporal stability via MD. AF2-based modeling suggests that the engagement principles we deduce for the model SP^L11^ can be generalized to many physiological SPs. Their binding mode is characterized by a 5-residue β-strand c-region and an h-region, which is significantly shorter than TM helices. Our combined findings suggest that the SPC-A-SP interaction is characterized by three major features (Fig. 4C): (A) The h-region of the SP is stabilized in the locally thinned transmembrane window, with contacts to both the surrounding lipids and the helical gear formed between Sec11A’s CTS and TM1. Our MD data show that both the membrane thinning and the angle at which the h-region is accommodated in the TM window are variable to a certain extent. The NTS functions as a lid, securing SP engagement with the CTS. (B) The transition from the helical h- to the β-strand c-region is structurally defined by the narrow gate framed by the Sec11A gatekeeper amino acids Thr153 and Trp36. (C) In the adjacent CBG, the P3 residue is positioned in the S3 pocket, and the P1 residue is accommodated by the S1 pocket, while the P2 and P4 residues experience no such constraints.

These structural features allow for variation of h-region length in the TM window, and the n-region is largely unrestrained. The SPC-A^S56A^-SP^L11^ structure suggests that c-regions must comprise at least five residues to span the distance from the active site to the transition gate. Further experimental studies will be required to clarify binding of selected SPs that cannot be modeled with high confidence. The structure also does not explain the association of substrates that deviate from the consensus architecture of SPs. Viral proteins with TM regions upstream the cleavage site, as well as mature TM proteins as part of quality control^3–5^ and transcription signaling^6^, await experimental clarification.

## Methods

### Design and cloning of expression constructs

All subunits of SPC-A were expressed from a single vector as described previously^1^. Sec11A was C-terminally tagged with a TEV-cleavable eGFP-TwinStrep-HA tag. The idealized SP^L11^ (MIKKKKLLLLLLLLLLLVPSAYA PYGSPGGSGGGSA) with a TEV-cleavable mCherry-3xFLAG-10xHis tag was cloned into a pUPE2961 vector backbone using Gibson assembly (New England Biolabs). All constructs were evaluated by sequencing. The residues of the idealized SP were chosen by residue frequency analyses of all SPs. ^P2^Tyr was chosen as a bulky handle that can be readily identified in cryo-EM maps even at moderate resolution.

### Analysis of SP^L11^ function

DNA sequences encoding SP^L11^-Prolactin, PreProlactin and Prolactin were cloned into the pcDNA3.1^+^ mammalian expression vector. HEKF 293-F cells (Thermo Fisher Scientific, R79007) were cultured in suspension in FreeStyle™ 293 Expression Medium (Thermo Fisher Scientific, 12338026) at 37 °C in a humidified incubator with 5% CO₂ and agitation at 120 rpm. Cells were passaged twice weekly, and cell density was maintained between 2.0 × 10^5^ and 3.0 × 10⁶ cells/mL

Twenty-four hours prior to transfection, cells were diluted at a concentration of 0.6 × 10⁶ cells/mL. For 30 mL of culture, 15 μg of plasmid DNA was diluted in Opti-MEM and combined with linear polyethylenimine (PEI; Polysciences) at a DNA:PEI ratio of [1:3] (w/w). The mixture was incubated at room temperature for (10–15) min to allow complex formation before being added dropwise to the cell suspension. Transfected cells were cultured under standard conditions as described above.

Cells and culture supernatants were harvested 48 h post-transfection by centrifugation (500 g, 5 min, 4 ˚C). The supernatant containing secreted proteins was collected, and the cell pellet was washed with ice-cold PBS (three times with ice-cold PBS, at 500 g, 5 min, 4 ˚C). Cells were resuspended in 1.5 mL of lysis buffer (10 mM HEPES pH 7.4, 250 mM Sucrose, 2 mM NaCl, 110 mM KoAc) supplemented with a protease inhibitor cocktail (Roche). Lysis was performed using an Isobiotec Cell Homogenizer (Isobiotec; 16 passes, 14 μm clearance) on ice. The lysate was clarified by centrifugation (1500 g, 3–4 × 10 min, 4 ˚C). Both the clarified lysate and culture supernatant were subjected to downstream immunoblotting analysis against prolactin (Thermo Fisher Scientific, PA5-79873).

### Protein production and purification

The construct coding for the SPC-A apo-enzyme was expressed as described previously^1^. For generation of the SPC-SPL11 complex structure, SPC-A and SPL11 were expressed at a molar ratio of 3:1. All constructs were transiently expressed for ~48 h in suspension HEK 293-E+ cells by ImmunoPrecise Antibodies (Europe) BV (Utrecht, the Netherlands) using 0.5 mg total vector DNA per L cell culture. The final cell densities ranged between 1–2 million cells per mL. All subsequent steps were performed at 4 ˚C unless stated otherwise. Cells were pelleted by centrifugation at 500 g and washed three times with ice-cold PBS to remove biotin from the expression medium. The resulting cell pellets were flash-frozen and stored at −80 ˚C until further use.

SPC-A holoenzyme was purified in PMAL-C8 as described previously^1^. SPC-A-SP^L11^ complexes were purified using a modified version of the protocol: Cell pellets from 4 L culture medium were thawed in 35 mL lysis buffer per L cell culture [50 mM HEPES pH 7.8 100 mM NaCl, 10% (v/v) glycerol, 10 mM imidazol, 0.7 μg/mL DNase I, 1% (w/v) DDM/CHS (1%/0.2%)] and incubated 1.5 h at 4 ˚C in a rotating wheel. One cOmplete inhibitor tablet (Roche, containing EDTA) per 35 mL was added during cell lysis. Cell debris was pelleted by ultracentrifugation at 100,000 g for 20 min in a fixed-angle rotor. The resulting supernatant was immobilized on 5 mL Ni-sepharose excel resin (Cytiva) using a pump with a flow rate of 1 mL/min, washed with 20 CV of buffer A [20 mM HEPES pH 7.8 85 mM NaCl, 10% (v/v) glycerol, 10 mM imidazole, 0.0174% (w/v) DDM/CHS (1% / 0.2%)], and eluted with ~10 CV buffer B [20 mM HEPES pH 7.8 85 mM NaCl, 10% (v/v) glycerol, 200 mM imidazole, 0.0174% (w/v) DDM/CHS]. The eluate was directly applied to 2 mL pre-equilibrated Streptactin 4Flow beads (IBA) in a gravity flow column, and the immobilized sample was washed with 20 column volumes (CV) wash buffer [20 mM HEPES pH 7.8, 85 mM NaCl, 1 mM EDTA, 10% (v/v) glycerol, 0.0174% (w/v) DDM/CHS]. Retained SPC was eluted with 10 CV elution buffer (100 mM HEPES pH 7.8, 85 mM NaCl, 1 mM EDTA, 10% (v/v) glycerol, 0.0174% (w/v) DDM/CHS, 50 mM biotin). The flowthrough was collected, concentrated, and applied on a Superose 6 increase 10/300 column (GE Healthcare) for size exclusion chromatography, equilibrated with size exclusion buffer (10 mM HEPES pH 7.8, 85 mM NaCl, 1 mM EDTA, 0.0174% DDM/CHS) (Figure S2E). Peak fractions were combined and concentrated to 7.75 mg/mL for cryo-EM sample preparation.

### Cryo-EM sample preparation

Grids for the SPC-A apo holoenzyme were prepared as described previously^1^, at 4 mg/mL with the addition of 1.5 mM fluorinated, fos-choline (FFosC, Anatrace) directly before vitrification. Similarly, SPC-A in DDM/CHS grids were prepared with both the presence of the surfactant and without. SPC-A^S56A^-SP^L11^ complexes were prepared without a surfactant at a concentration of 7.75 mg/mL. 3 µL samples were applied to freshly glow-discharged Cu 200 Holey Carbon R2/1 grids (Quantifoil). In both cases, grids were flash-frozen using a Vitrobot Mark IV (Thermo Fisher Scientific) with 595 blotting paper (Ted Pella) at 4 ˚C, 100% humidity, blot force of 0 for 4 s and a liquid ethane/propane mixture.

### Cryo-EM data collection

Cryo-EM data of SPC-A-SP^L11^ and the apo-SPC-A (DDM/CHS) were collected on a Titan Krios (Thermo Fisher Scientific) equipped with an E-CFEG, Falcon 4i detector and Selectris X energy filter. Movies containing 1530 EER frames for both datasets were acquired using EPU 3.1 at a nominal magnification of × 165,000 corresponding to an image pixel size at the detector of 0.73 Å per pixel. The total dose of 60 electrons/Å^2^ was applied to the movies over an exposure time of 4.99 s, corresponding to a dose-rate of 6.4 electrons per pixel per second (or 12 electrons per Å^2^/s). Movies were acquired with target defocus values of −0.8, −1.0 and −1.2 μm. Autofocus was performed only once in the central hole of each aberration free image shift hole cluster of 12 μm in radius. Three images per hole were acquired using a 700 nm fringe-free beam. Apo-PMAL-C8 was collected on a 300 kV Titan Krios microscope (Thermo Fisher Scientific) equipped with a post-column energy filter (slit width 20 eV) and a K3 direct electron detector (Gatan). EPU (Thermo Fisher Scientific) was used for automated data collection in counting mode. Movies were acquired in 45–50 frames at a pixel size of 0.337 Å/px in super-resolution mode, with a dose rate of 23.76 e-/px/s (measured in an empty hole without ice), and a total dose of 60 e-/Å^2^. Defocus values ranged between −0.7 and −3.1 μm. Data quality was monitored in real time using Warp.

### Image processing

Collected movie stacks for SPC-A-SP^L11^ were manually inspected and imported into cryoSPARC v.3.3.1^36^ where patch motion and CTF correction were executed. Movies exhibiting an estimated resolution worse than 5 Å, high astigmatism and a motion over 20 Å were discarded, leading to a final set of 8504 movies. A total of 1,743,807 particles were template picked from 8504 movies, utilizing 2D-templates generated through blob-picking from a subset of initial micrographs. The particles were then extracted in a box size of 360 pixels, at three-fold binning. Subsequently, the extracted particles were subjected to three rounds of 2D-classification to remove junk particles. An ab-initio model exhibiting good model features was generated and refined with multiple heterogeneous refinements serving as an initial model for 3D classification. The resulting 446.574 particles from the best class were re-extracted in an unbinned box size of 360 pixels and subjected to another round of refinements. Initial refinement using the non-uniform refinement tool yielded a 3.54 Å resolution-map. A final round of 3D-classification and a local refinement using a stringent mask was performed resulting in the 2.5 Å-resolution map that was used for model building and model generation (Supplementary Table 1). The datasets collected for apo SPC-A in DDM/CHS and PMAL-C8 were processed analogously.

### Atomic model building and refinement

The initial atomic model of SPC-SP^L11^ was generated using ModelAngelo v.1.0.1, which utilized the FASTA sequence obtained from our previously published cryo-EM structure of SPC Sec11A (PDB ID: 7P2P). Following the generation of the initial model, initial flexible fitting was conducted in COOT v.0.9.8.3^37^ through manual refinement of the entire peptide chain. Subsequently, real space refinement was performed in Phenix v.1.20.1-4487^38^. This iterative process involving COOT and Phenix was repeated to minimize Ramachandran angle outliers and rotamer outliers, optimize the fit to density, and eliminate steric clashes. To validate the final model, EMRinger^39^ and MolProbity^40^ were employed. All figures were prepared using UCSF ChimeraX^41^.

### Initial model generation for Molecular Dynamics simulation

The initial protein structures were generated using a local installation of AlphaFold2-Multimer ^23^and validated against the Cryo-EM structure. The protonation state of titratable residues was assigned using PROPKA 3.0^42,43^ at pH 7.0 through the PDB2PQR webserver^44^ and through visual inspection of charged residue location.

The CG model of the protein was generated with the newest version (v0.15.0) of Martinize2^45,46^. Sidechain fixes were applied along with elastic networks to each monomer in the complex, with a distance cutoff of 0.8 nm and a force constant of 700 kJ mol^−1^nm^−2^, with the exception of the peptide in question. Additional harmonic bonds were applied across the monomers to stabilize the complex further. These additional were placed between MET 1 in chain A and ASP 49 in chain C, ILE 81 in chain C and ARG 16 in chain A, TRP 80 in chain B and GLU 110 in chain D and THR 55 in chain B and HIS 131 in chain A with force constants of 70, 30, 70, 70 kJ mol^−1^nm^−2^, respectively.

### Setup of coarse-grained MD simulations

All the CG simulations were performed with the Martini 3 Force Field^47^. The SPC complex was embedded into a symmetric ER membrane model using INSANE^48^, composed of POPC:POPE:POPS:Cholesterol:PI(3,4)P2 with the ratios 44:26:4:15:11^15^ in a cubic box of 15 × 15 × 15 nm. The normal of the bilayer along with the principal axis of the SPC complex were aligned with the z-axis. All the systems were solvated with water and 0.15 M NaCl.

The systems were minimized by using steepest descent with 6000 steps while applying position restraint to the backbone beads and using bonds instead of constraints for the PI(3,4)P2 lipids^49^. Following the minimization, two steps of relaxation were done with an increased time step from 5 to 10 fs for 1 ns each. During the first step, harmonic bonds were used instead of constraints for the PI(3,4)P2 lipids.

The settings for the CG simulations were the recommended settings for “new” martini simulations parameters^50^. For the relaxation steps, the temperature and pressure were kept constant at 310 K and 1 bar, respectively, using the Berendsen thermo- and barostat^51^, with a semi-isotropic pressure coupling and a compressibility of 3 × 10^4 ^bar^−1^. The protein, membrane, and solvent were all coupled separately.

For the production run, five repeats of each 20 µs were performed, using a timestep of 20 fs, each initiated using a random seed. The temperature was kept constant at 310 K using the velocity rescale thermostat, while the pressure was maintained with the Parrinello-Rahman barostat^52^. All the simulations were done using the software Gromacs version 2020^53^.

A reference system of the protein complex in a pure POPC membrane was also constructed and simulated for comparison. The same protocol as described above was used.

### Setup of atomistic MD simulations

The CG model of the SPC complex was embedded into a bilayer and run as described above for 1 µs production run, with the exception of having position restraints of the backbone beads throughout relaxation and production run. This was done to equilibrate the membrane, before changing the resolution to atomistic.

The last frame of the simulation was back-mapped into atomistic resolution using CG2AT^54^. The system was then minimized and equilibrated, followed by five repeated production runs, each being 1 µs long, using a timestep of 2 fs. The CHARMM36m Force Field^55^ was used along with Gromacs version 2020. The systems were all solvated with the TIP3P water model^56^ and 0.15 mM NaCl. The temperature and pressure were both kept constant at 310 K and 1 bar using the velocity rescale thermostat ^57^and the Parrinello-Rahman barostat^52^, respectively, with a compressibility of 4.5 × 10^−5 ^bar^−1^ with a coupling time constant of 5 ps. The electrostatics were described using Particle Mesh Ewald^58^ with a cut-off value of 1.2 nm. Van der Waals interactions were switched to zero between 1.0 and 1.2 nm. The neighbor list was updated every 20 steps. The LINCS algorithm^59^ was used to constraint all bonds involving hydrogens. Minimization and relaxation were based on the CHARMM-GUI protocol^50,60^. Simulations of the human Integrin alpha-IIb SP (Uniprot ID: P08514) were performed using the same approach, constructing and relaxing the system at CG resolution after which it was converted to atomistic resolution and simulated.

### Analysis of MD trajectories

Two approaches were used for investigating contacts between SPC and the peptide in question.

For comparison across atomistic and CG resolution, contact frequencies were calculated, considering a contact if any atom or simulation bead is within 6 or 7 Å for atomistic and CG, respectively. This was done using the Gromacs tool gmx pairdist^53^, and a python script utilizing the package numpy^61^.

Additionally, for the atomistic simulations, the python package ProLif^62^ was used to analyze protein-peptide interactions in depth. All interactions except Metal-Acceptor and Metal-Donor interactions were tracked. All simulations were split into three blocks (each 333 ns, dividing the 1 μs simulation into thirds), and a protein-peptide contact was considered as conserved if it was present above 60% of the simulation time in three out of five replicas per block and in at least two out of three blocks.

The thinning of the membrane in the TM window was calculated as the difference in number density of PO4 beads in the TM vs the bulk. The TM window was defined as 8 Å around resid 2 to 27 in chain B, resid 27 to 52 and resid 162 to 190 in chain A and resid 111 to 143 in chain C. A reference membrane of the same composition was simulated for 1µs for the bulk density value (Supplementary Fig. 11). For the apo CG systems three different cutoffs were tested, at 6 Å, 8 Å, and 10 Å, respectively, with the same trend visible across all three values: for the 6 Å cutoff, no lipid heads: 12.9 ± 0.2 and 26.6 ± 1.6% thinning, for the 8 Å cutoff, no lipid heads: 17.9 ± 0.3, and 25.5 ± 1.4% thinning, and for the 10 Å cutoff, no lipid heads 25.5 ± 0.4, and 22.8 ± 1.2%, respectively. By selecting the 8 Å cutoff, whole beads are ensured to be selected while being highly selective to the TM window. An overview of the simulated systems can be found in SI (Supplementary Table S2).

The tilting angle of the peptides was calculated using a python script based on MDAnalysis^63,64^ and numpy. The tilt angle was calculated as the angle between the normal of the membrane (z-axis) and the vector described from the three first and three last residues in the transmembrane helix (Supplementary Fig. 7).

For checking convergence of the simulations RMSD and RMSF were calculated of the various units in the protein complex (Supplementary Figs. S12, S13). The RMSF of SPC-A was calculated using the backbone for 250 ns atomistic simulation and 1 µs coarse grained simulation. The same starting reference structure from atomistic was used. The RMSD of the SPC complex was calculated using the backbone C-alpha atoms or backbone beads. The complex was first fitted to the experimental structure using only SPC-A.

The python package MDAnalysis (release 2.10.0) was used to calculate both the RMSD and RMSF properties^63,65–68^. The RMSF plot comprised running averages over 10 ns for both CG and AA.

### Modeling of human signal peptides bound to Sec11A

A custom automated analysis pipeline was developed using Python (v3.11) and the Biopython library^69^ for SP modeling analysis.

Geometric Definition of the Transition Gate: AlphaFold2-Multimer^23^ models of N = 412 SPs^15^ annotated *Homo sapiens* signal peptides in complex with the Sec11A catalytic subunit of the SPC-A (Uniprot ID: P67812) were generated and analyzed to identify the SP region transition gate between the “gatekeeper” residues. The spatial reference for the gate, denoted as ${{{\bf{M}}}}_{{{\rm{gate}}}}$, was defined as the geometric midpoint between the side-chain centroids of the conserved gatekeeper residues, Trp36 and Thr153 (Sec11A numbering). The centroid for each gatekeeper residue was calculated as the arithmetic mean of the coordinates of all side-chain atoms (excluding backbone N, C, and O atoms). For Glycine residues, the Cα coordinate was used as the centroid. To spatially register the peptide within this gate, we identified a specific “anchor residue” (position 0) for each model. With ${{{\bf{r}}}}_{i}$ defining the coordinate vector of the Cα atom for the $i-{th}$ residue of the signal peptide (Chain B) the anchor index $k$ was determined by minimizing the Euclidean distance to the gate midpoint:

1 $${k={{{\rm{argmin}}}}_{i}\parallel {{\bf{r}}}}_{i}-{{{\bf{M}}}}_{{{\rm{gate}}}}\parallel$$

This residue $k$ defined the center of the trans-gate window for all subsequent analyses.

Model Filtering and Quality Control: To ensure biological relevance and structural quality, models were subjected to a strict four-step filtering protocol: 1. Confidence Filter (pLDDT): models were first evaluated for local confidence. Only models exhibiting a mean predicted Local Distance Difference Test (pLDDT) score $\ge$ 70 for the trans-gate window (anchor residue $\pm$ 3 residues) were retained. 2. Topology Validation (Orientation)**:** Peptide insertion topology was validated by calculating the Euclidean distances from the peptide N- and C-termini to a reference coordinate on the cytosolic side of the enzyme (Sec11A C-ter Glu179 side-chain centroid). Models were required to exhibit the correct membrane insertion orientation, defined as the N-terminus being spatially closer to Glu179 than the C-terminus ${d}_{{{\rm{N}}}-{{\rm{Glu}}}179} < {d}_{{{\rm{C}}}-{{\rm{Glu}}}179}$. 3. C-region Length: To ensure the presence of a sufficient peptide segment extending beyond the transition point for potential S1 pocket engagement, we required a minimum tail length of 5 residues downstream of the anchor position (${L}_{{{\rm{tail}}}}\,\ge \,5$). 4. Hydrophobic Pocket Proximity (Centroid-Based): To assess the engagement of the peptide with the enzyme’s S1 hydrophobic pocket, we defined a coarse-grained distance metric, ${d}_{{{\rm{pocket}}}}$. S1 Pocket Reference (${{{\bf{C}}}}_{{{\rm{pocket}}}}$): Defined as the geometric centroid of the side-chain atoms of the four S1 hydrophobic pocket residues: Val52, Ile57, Phe61, and Ile95. Peptide Reference (${{{\bf{C}}}}_{{{\rm{pep}}}}$): Defined as the geometric centroid of the side-chain atoms of the C-terminal P1 residue of the signal peptide. Models were classified as potentially “entering” or “docked” if the distance satisfied $= \,\parallel {{{\bf{C}}}}_{{{\rm{pep}}}}-{{{\bf{C}}}}_{{{\rm{pocket}}}}\parallel \,\le 8.0\,{{{\AA }}}$, in line with definitions of “encounter complex” formation^70^. This threshold accounts for the physical volume of the pocket side chains and defines a boundary for loose hydrophobic association appropriate for centroid-based filtering. After strict application of all 4 quality control filters, *N* = 92 (22.3%) Sec11A-SP complex models satisfied all criteria and were considered as robust. Batch processing of PDB files, geometric calculations, and data aggregation were performed using the NumPy^61^ and Pandas^61^ libraries. Distributions of model confidence were visualized using Seaborn^71^ and Matplotlib^72^.

Structural Alignment and Positional RMSD Analysis: Comparative structural analysis was performed using UCSF ChimeraX (v1.11). To evaluate the conformational fidelity of predicted complexes against the experimental reference, a specialized alignment protocol was implemented. For each predicted model, the Sec11A scaffold (Chain A) was globally aligned to the reference scaffold using the matchmaker algorithm, which employs a Needleman-Wunsch alignment followed by a least-squares fit of Cα coordinates. The positional deviation of the modeled SP (Chain B) was quantified by calculating the Euclidean distance for each Cα atom relative to the reference SP. Given the biological importance of the c-region, residues were indexed from the C-terminus (denoted as ${P}_{1},{P}_{2},\,{P}_{3},$… ${P}_{22}$). For each position *i* in each model, the positional deviation was defined as the minimum distance to the corresponding Cα atom in the reference SP to account for local shifts, calculated as:

2 $${{d}_{i,\,j}={\min }_{k}\parallel {{\bf{v}}}}_{i,j}-{{{\bf{r}}}}_{k}\parallel$$

where ${{{\bf{v}}}}_{i,j}$ represents the Cartesian coordinates of the Cα atom at position i in the $j$-th predicted model, and ${{{\bf{r}}}}_{k}$ represents the coordinates of the k-th Cα atom in the experimental reference SP. Statistical Scoring and Confidence Metrics: To assess the reliability of the predicted ensembles, Predicted Local Distance Difference Test (pLDDT) scores were extracted directly from the B-factor columns of the model PDB files. The pLDDT serves as a per-residue measure of local confidence, where scores $\ge$ 90 indicate high-accuracy side-chain orientation and 70–90 indicate reliable backbone modeling. For both positional deviation and pLDDT, ensemble statistics were computed across all accepted models (*N* = 92), and the mean value and standard deviation (SD) for each residue position were calculated. Visualization and Data Integration were performed using a custom Python pipeline (Python v3.11) employing the Matplotlib and Pandas libraries.

### Sequence alignment and gatekeeper evolutionary conservation analysis

Orthologs of the *H. sapiens* Sec11A subunit (UniProt ID: P67812) were retrieved from the UniProt database^73^ (Access Date: 15-01-2026). To ensure a comprehensive analysis of eukaryotic evolutionary conservation, a diverse set of 20 representative species was selected. The set included distinct classes of Vertebrata (Mammalia, Aves, Reptilia, Amphibia, and Actinopterygii), as well as representative Invertebrata (e.g., *Drosophila melanogaster*, *Caenorhabditis elegans*) and Fungi (*Saccharomyces cerevisiae*, *Schizosaccharomyces pombe*). Multiple sequence alignment (MSA) was performed using MUSCLE^74^ with default parameters, implemented within the Jalview v2.11.5.1 desktop environment^75^, and the resulting alignment was manually inspected for quality. Conservation scores were calculated based on physicochemical property grouping (BLOSUM62 score) and percentage identity.

### *Reporting summary*

Further information on research design is available in the Nature Portfolio Reporting Summary linked to this article.

## Supplementary information

Supplementary Information

Reporting Summary

Transparent Peer Review file

## Source data

Source Data 1

Source Data 2

## Data Availability

Data generated in this study have been deposited at the Electron Microscopy Data Bank under accessions EMD-54010 and Protein Data Bank ID 9RJB for the SPC-A^S56A^SP^L11^ and EMD-54011 and PDB ID 9RJC for the apo SPC-A. The Alphafold2-multimer models generated for the analysis, along with the raw MD trajectory data and additional plots are archived in Zenodo under [10.5281/zenodo.18299233]. Source data are provided with this paper.

## Code availability

The code used for structural filtering and positional deviation analysis is available on GitHub at https://github.com/SBC-Utrecht/Sec11A-Signal-Peptide-Analysis. The version of the code used for the final analysis is archived on Zenodo under 10.5281/zenodo.18620566 [10.5281/zenodo.18620566]. All code is released under the MIT License.
