# Supplementary material for: Structural basis of signal peptide recognition by the signal peptidase complex
Source: Nat Commun. 2026 May 22;17:6748. doi: 10.1038/s41467-026-73423-3 (PMC13385932; doi:10.1038/s41467-026-73423-3)
Supplement: Supplementary file 1 — Supplementary Information [file 41467_2026_73423_MOESM1_ESM.pdf]

Supplementary Information for

Structural basis of signal peptide recognition by the signal peptidase complex

A. Manuel Liaci[1,+], Dimitrios Vismpas[1,+], Lisbeth R. Kjølbye[2], Ioannis Skalidis[1], Gilberto P. Pereira[2,3,4,5], Adrian Fujiet Koh[6], Mariska Gröllers-Mulderij[1], Abhay Kotecha[6], Paulo C. T. Souza[2,3,4], Friedrich Förster[1*]

Affiliations:

1: Structural Biochemistry, Bijvoet Centre for Biomolecular Research, Utrecht University, Universiteitsweg 99, 3584 CG, Utrecht, The Netherlands.

2: Molecular Microbiology and Structural Biochemistry, CNRS UMR 5086 and Université Claude Bernard Lyon 1, Lyon, France.

3: Laboratoire de Biologie et Modélisation de la Cellule, CNRS, UMR 5239, Inserm, U1293, Université Claude Bernard Lyon 1, Ecole Normale Supérieure de Lyon, Lyon, France.

4: Centre Blaise Pascal de Simulation et de Modélisation Numérique, Ecole Normale Supérieure de Lyon, Lyon, France

5 : present address : Zymvol Biomodeling S. L., C/ Pau Claris, 94, 3B, 08010 Barcelona, Spain

6: Thermo Fisher Scientific, Achtseweg Noord, 5651 GG, Eindhoven, The Netherlands

[+]: These authors contributed equally

[*]Correspondence to: f.g.forster@uu.nl

Table of Contents

Supplementary Figures ***3***

Supplementary Tables ***23***

Uncropped gels ***27***

Supplementary Figures

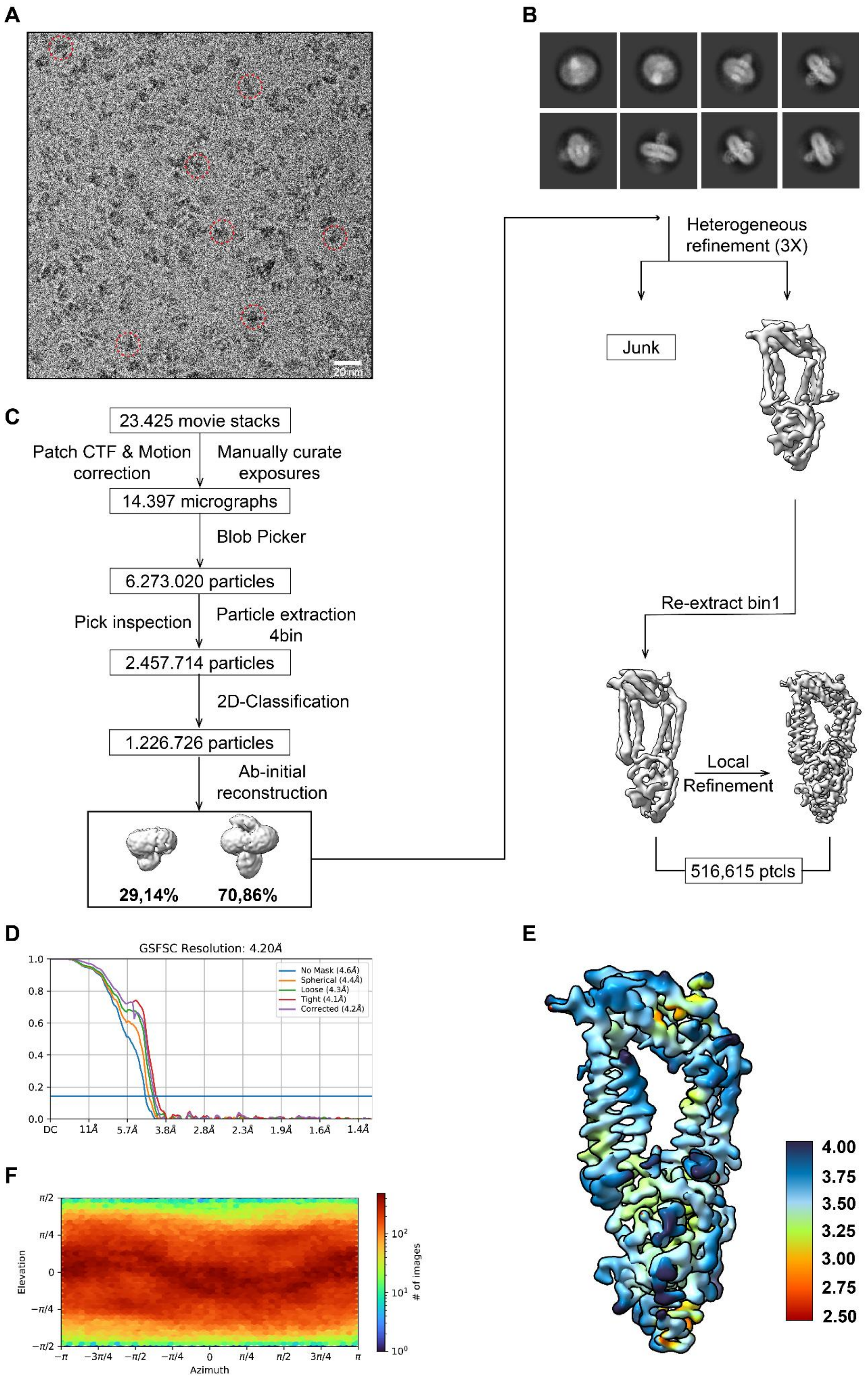

Supplementary Figure 1: Cryo-EM processing and 3D-reconstruction workflow for the apo-SPC. A. Representative motion corrected micrograph (K3). Example apo SPC-A particles are indicated in red B, Indicative 2D-class averages. C, Schematic depiction of the used workflow for the image processing. D, Gold-Standard FSC (0.143 cutoff). E, Local resolution map of the final 3D-reconstruction of the complex. F, Angular distribution calculated in cryoSPARC for particle projections.

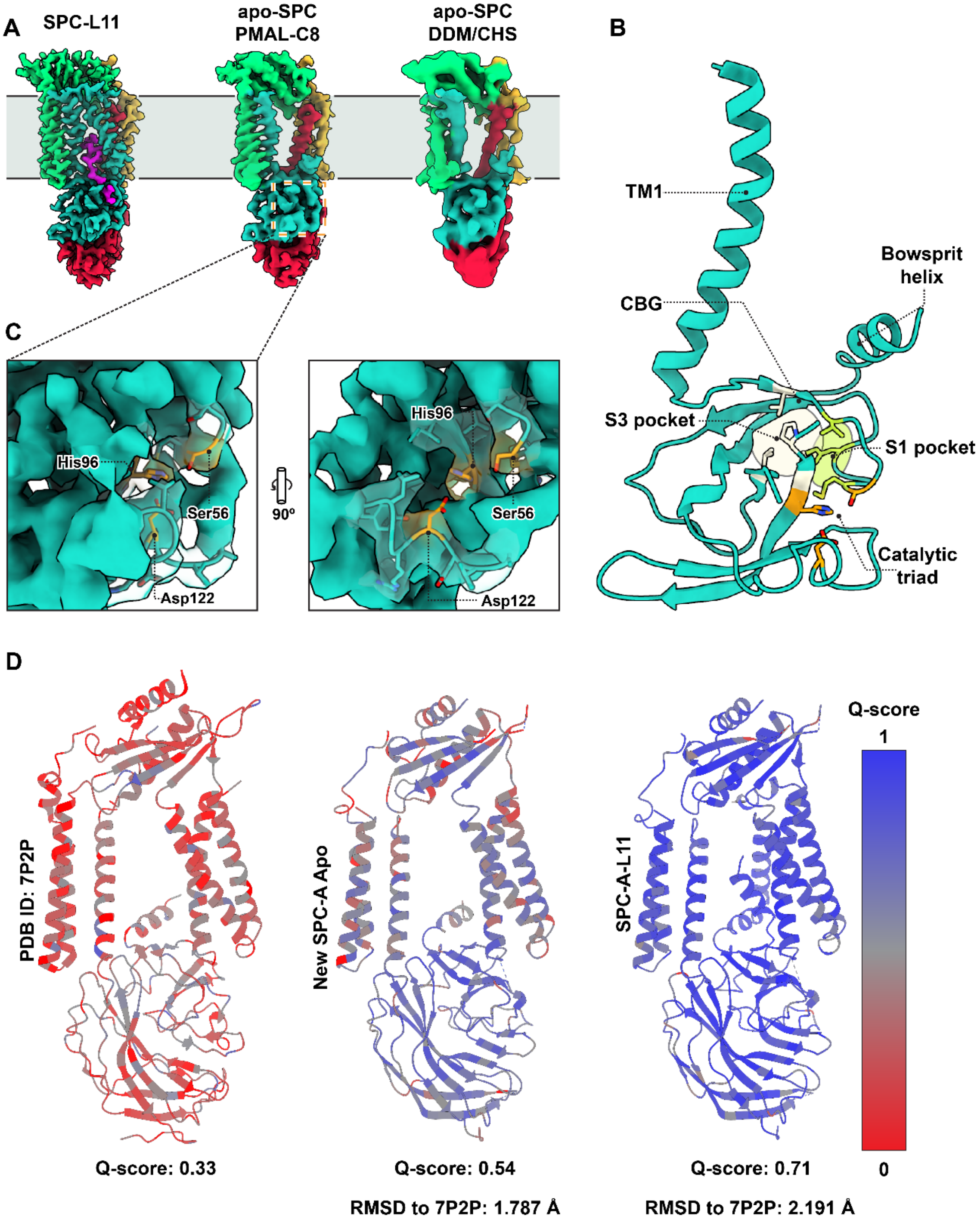

Supplementary Figure 2: Comparison of SPC-A Cryo-EM structures. A, EM-maps of the SPC-A^{S56A}-SP^{L11} complex, apo SPC-A in amphipol and apo SPC-A in DDM/CHS, respectively (colored as in Figure 1) **B**, Schematic overview of the catalytic subunit of apo SPC-A. **C**, The catalytic triad Asp-Ser-His rendered in orange (EM-density contour level 0.15). **D,** Q-scores of resolved residues for (apo)SPC-A models.

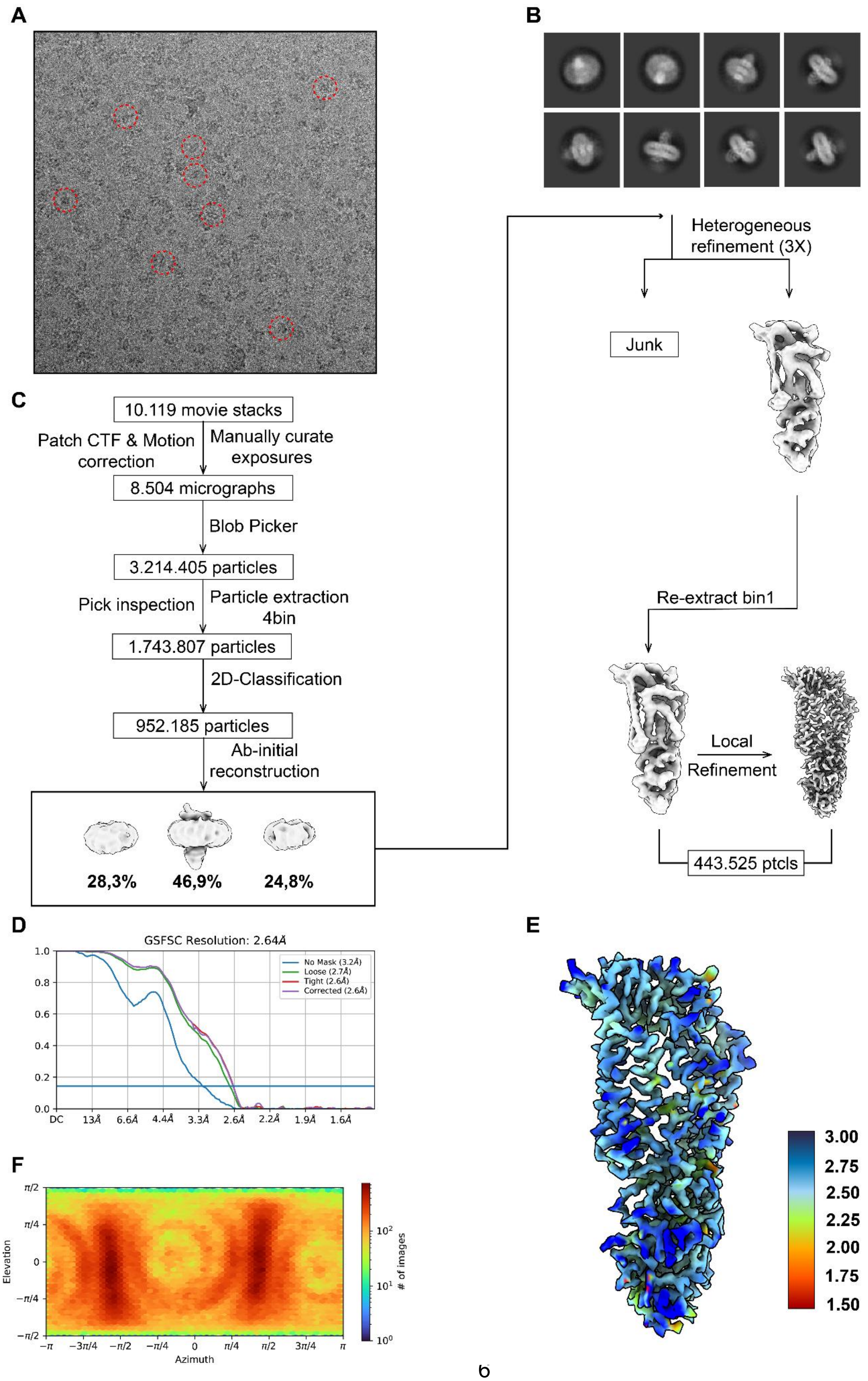
A
B
Heterogeneous
refinement (3X)
Junk
C
10.119 movie stacks
Patch CTF & Motion
correction
Manually curate
exposures
8.504 micrographs
Blob Picker
3.214.405 particles
Pick inspection
Particle extraction
4bin
1.743.807 particles
2D-Classification
952.185 particles
Ab-initial
reconstruction
28,3%
46,9%
24,8%
Re-extract bin1
Local
Refinement
443.525 ptcls
D
GSFSC Resolution: 2.64Å
No Mask (3.2Å)
Loose (2.7Å)
Tight (2.6Å)
Corrected (2.6Å)
1.0
0.8
0.6
0.4
0.2
0.0
DC
13Å
6.6Å
4.4Å
3.3Å
2.6Å
2.2Å
1.9Å
1.6Å
E
3.00
2.75
2.50
2.25
2.00
1.75
1.50
F
π/2
π/4
0
−π/4
−π/2
Elevation
−π
−3π/4
−π/2
−π/4
0
π/4
π/2
3π/4
π
Azimuth
# of images

Supplementary Figure 3: Cryo-EM processing and 3D-reconstruction workflow for the SPC-A^{S56A}-SP^{L11} complex. A, Representative motion corrected micrograph (Falcon IV). Example SPC-A^{S56A}-SP^{L11} particles are indicated in red B, Indicative 2D-class averages. C, Schematic depiction of the used workflow for the image processing. D, Gold-Standard FSC (0.143 cutoff). E, Local resolution map of the final 3D-reconstruction of the complex. F, Angular distribution calculated in cryoSPARC for particle projections.

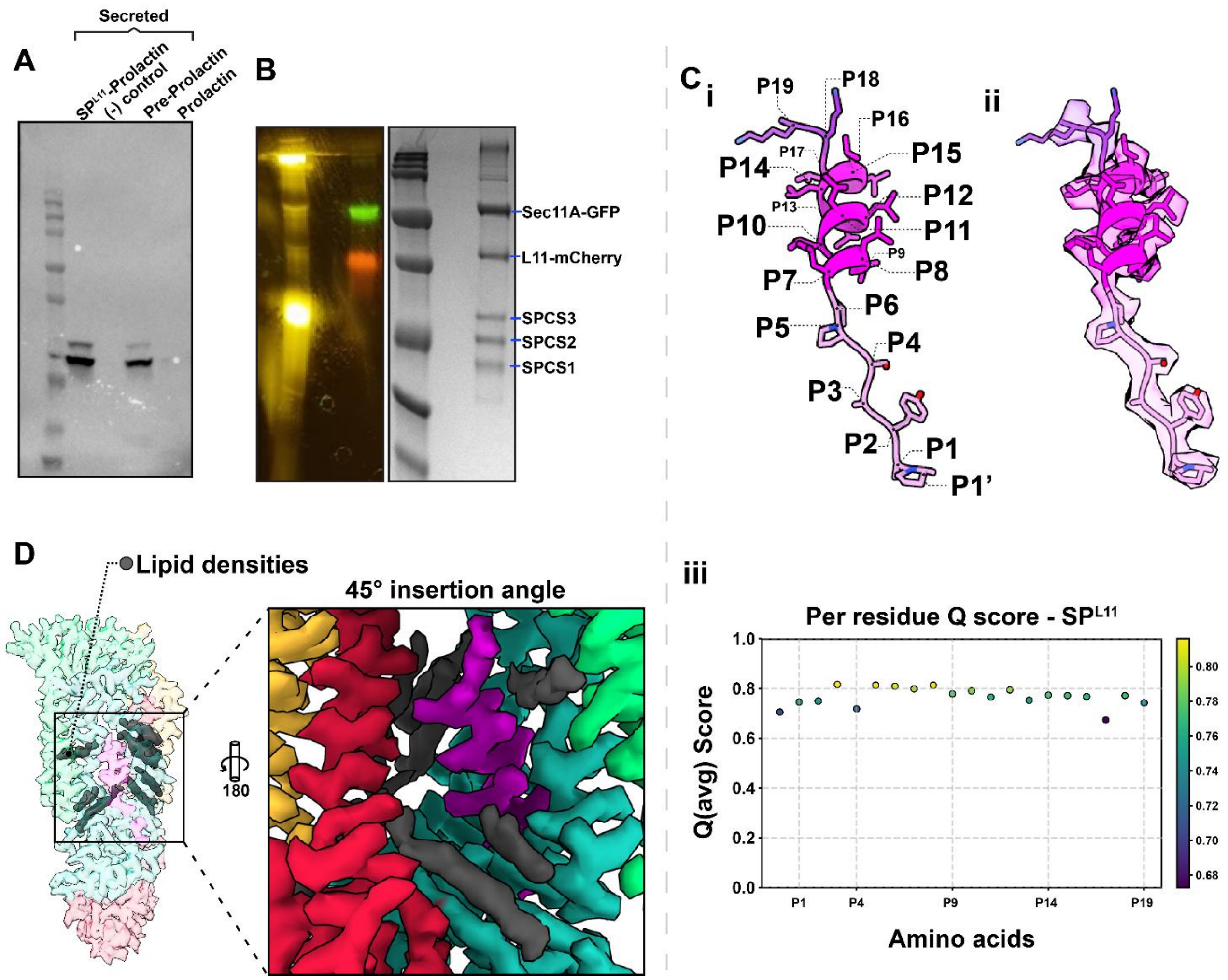

Supplementary Figure 4: SP^{L11} functional, biochemical and structural analysis. A, Western blot against secreted prolactin fused with SP^{L11} (SP^{L11}-Prolactin) its native SP (Pre-prolactin) and without any SP (Prolactin) transiently expressed in HEK-298 cells. The (-) is an empty vector control. B, Tris-Tricine SDS-PAGE of the complex purification. Left panel: Blue/green LED analysis visualizes the presence of SEC11A-GFP (green) and SP^{L11}-mCherry (red). C(i), SP^{L11} residue position naming. (ii), Cryo-EM map quality for SP^{L11} (average Q-score of modeled residues: 0.71). (iii) SP^{L11} per-residue Q-score plot. D, Lipid densities, highlighted with dark grey, (EM-density contour level 0.102) captured entering the transmembrane window with a 45° insertion angle.

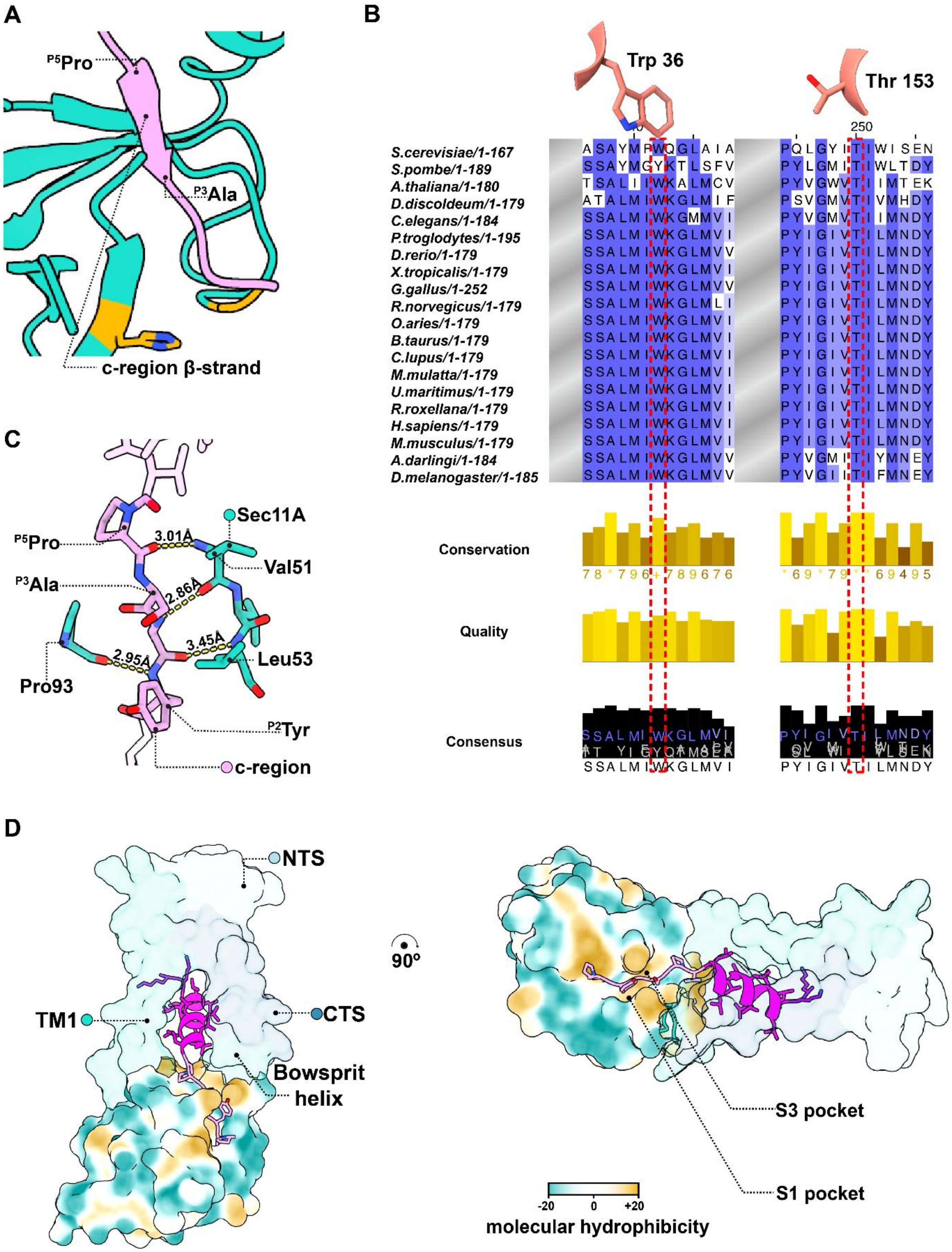

Supplementary Figure 5: SP^{L11} c-region stabilization in the CBG and evolutionary conservation of gatekeeper residues. A, (i) The SP^{L11} c-region adopts a β-strand conformation (H-bond energy cutoff in DSSP [79]: -0.18 kcal/mol). B, Evolutionary conservation of gatekeeper

residues Sec11A Trp36 and Thr153 for selected eukaryotic organisms. C, Hydrogen bonds (yellow) between Sec11A and SP^{L11}. Residues P2, P3 and P5 anchor the SP in the CBG. D, The Sec11A-SP^{L11} complex is illustrated as a surface model. CBG is highlighted while the NTS, TM1, bowsprit helix, and CTS are shown in lower opacity. Hydrophobic pockets S1 and S3 are indicated.

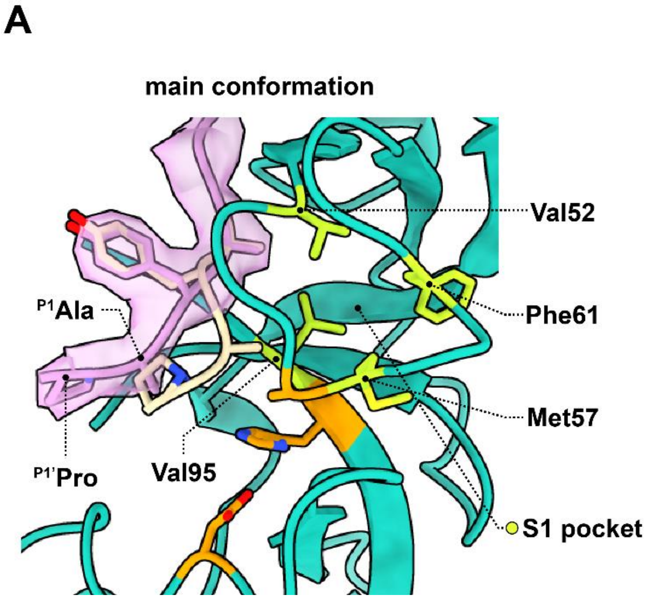

Supplementary Figure 6: Fractional occupancy of pocket S1 by ^{P1}Ala. A, Main conformation of experimentally resolved SP^{L11} (plum: EM-density contour level 0.111). The alternative SP model of the c-region conformation is annotated in light yellow. B, Alternative conformation of experimentally resolved SP^{L11} (light yellow: EM-density contour level 0.067) is highlighted with the sidechain of the ^{P1}Ala partially occupying pocket S1.

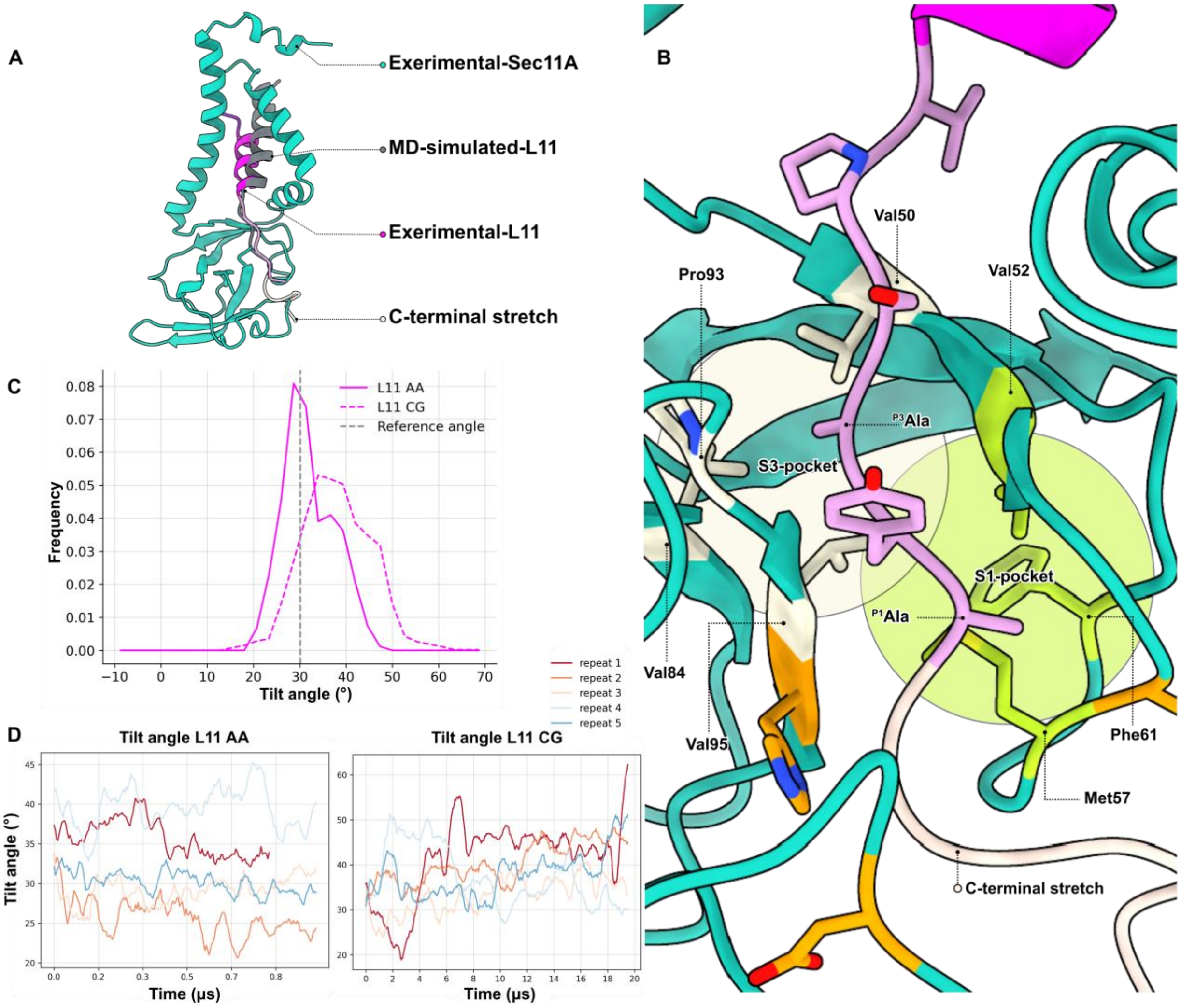

Supplementary Figure 7: Investigating the SPC-A^{S56A}-SP^{L11} complex by Molecular Dynamics simulations. A, Superposition of the experimental SP^{L11} (magenta) on the SP used for MD studies (gray), which also contains a C-terminal stretch (beige) not resolved in the experimental structure. B, Snapshot of the CBG during the simulation. S3 and S1 pockets are highlighted (similar to Figure 2C-D). Both $^{P3\&P1}$Ala enter the S3 and S1 pockets accordingly. C, Tilt angle distribution plot of SP^{L11} h-region compared to the ER-membrane. D, Tilt angle during simulation time for AA and CG systems.

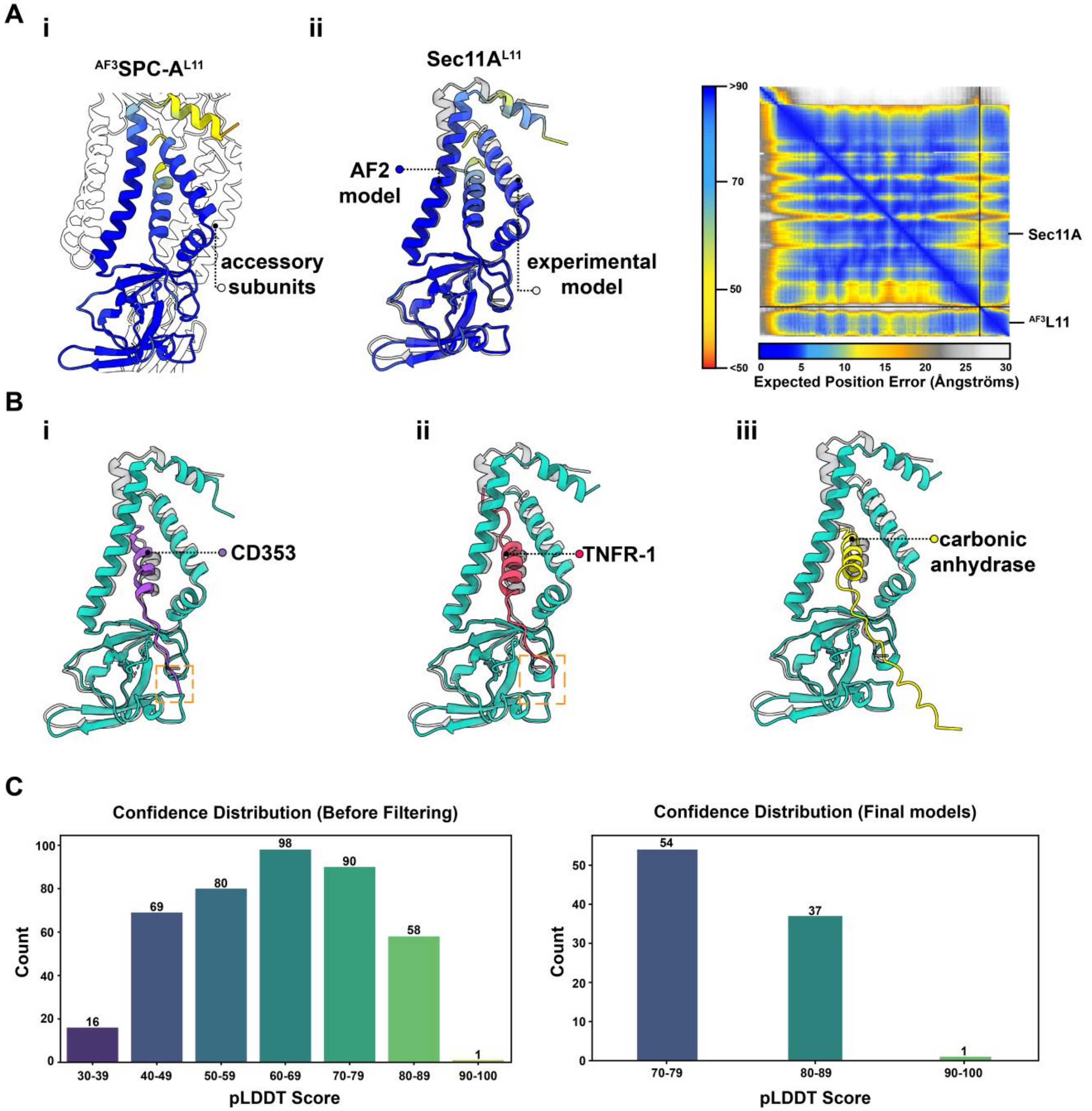

Supplementary Figure 8: Assessment of AlphaFold2 modeling. A, (i) AF2 model of SPC-A interacting with the SP^{L11}. Accessory subunits are blurred out (ii) AF2 model of SP^{L11} interacting with the catalytic subunit Sec11A only superposed on the experimental structure (Sec11A-SP^{L11}: dark grey). Quality estimation for AF2 prediction. Model representation is color-coded according to the local confidence estimated with the predicted local distance difference test (plDDT). Dark blue (>90) indicates high confidence whereas red (<50) represents low confidence regions. Interchain and interdomain Predicted Aligned Error (PAE, in Å), between all different residue pairs in a complex, showcases low expected error (blue). B, (i,ii) Examples of AF2 failure mode I. Inaccurately predicted AF2 models (plDDT<70), where SPs showcase a register shift relative to

the cleavage site (highlighted in an orange dotted box). (iii) AF2 failure mode II. Erroneous AF2 model, where the SP is inserted in the wrong topology (N-terminus near active site). C, AF2-multimer pLDDT score distribution. Left: pLDDT score distribution before filtering. Right: pLDDT score distribution of final valid models. Source data are provided as a Source Data 2 file and in Zenodo (DOI: 10.5281/zenodo.18299233).

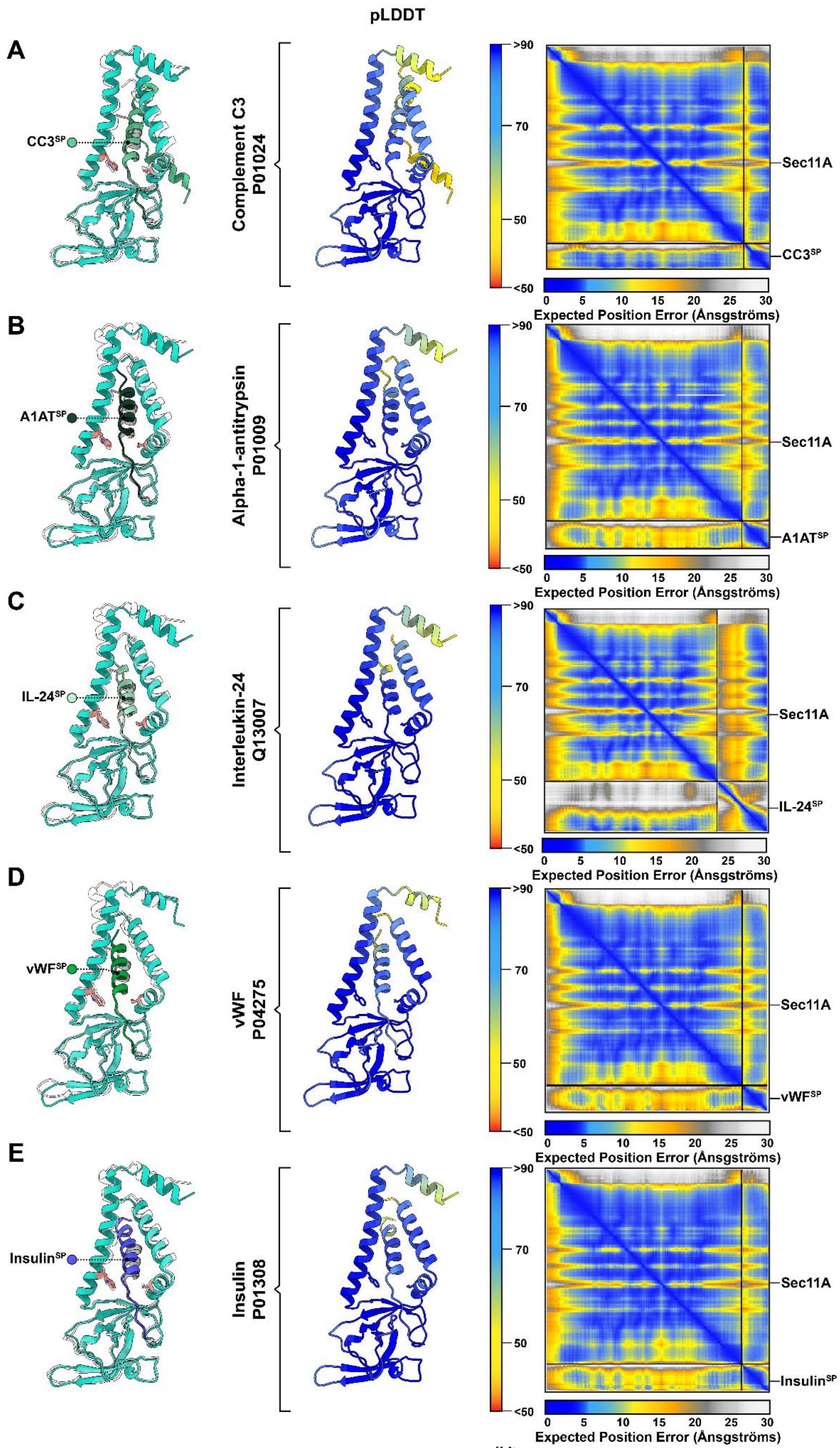
pLDDT
A
CC3^SP
Complement C3
P01024
>90
70
50
<50
Sec11A
CC3^SP
0 5 10 15 20 25 30
Expected Position Error (Ånsgströms)
B
A1AT^SP
Alpha-1-antitrypsin
P01009
>90
70
50
<50
Sec11A
A1AT^SP
0 5 10 15 20 25 30
Expected Position Error (Ånsgströms)
C
IL-24^SP
Interleukin-24
Q13007
>90
70
50
<50
Sec11A
IL-24^SP
0 5 10 15 20 25 30
Expected Position Error (Ånsgströms)
D
vWF^SP
vWF
P04275
>90
70
50
<50
Sec11A
vWF^SP
0 5 10 15 20 25 30
Expected Position Error (Ånsgströms)
E
Insulin^SP
Insulin
P01308
>90
70
50
<50
Sec11A
Insulin^SP
0 5 10 15 20 25 30
Expected Position Error (Ånsgströms)

Supplementary Figure 9: AlphaFold2 examples of SP-engagement. A-E: Five high-confidence Sec11A-SP models obtained using AF2 (A: $CC3^{SP}$, B: $A1AT^{SP}$, C: $IL\text{-}24^{SP}$, D: vWF^{SP}, E: $Insulin^{SP}$). Sec11A (teal) is superposed on the experimental structure (Sec11A: white, SP^{L11}: light grey). The confidence pLDDT estimations and the per residue Expected Position Error are displayed on the right.

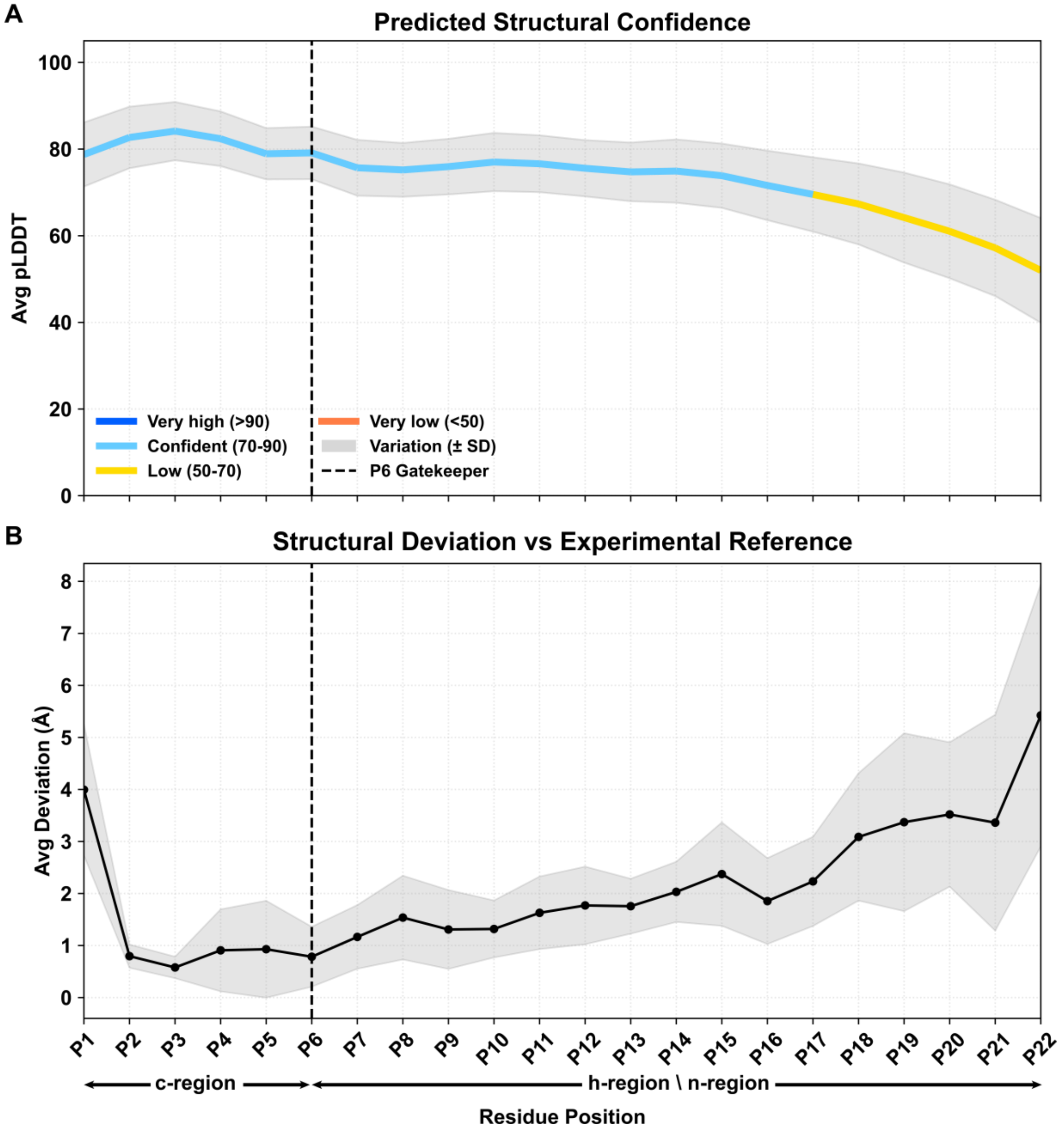

Supplementary Figure 10: Confidence and positional variability of 92 high-confidence SP models. A, Average pLDDT per SP position. B, Average RMSD per SP position compared to SP^{L11}. All models were structurally aligned to the experimental Sec11A structure. Source data are provided as a Source Data 2 file and in Zenodo (DOI: 10.5281/zenodo.18299233).

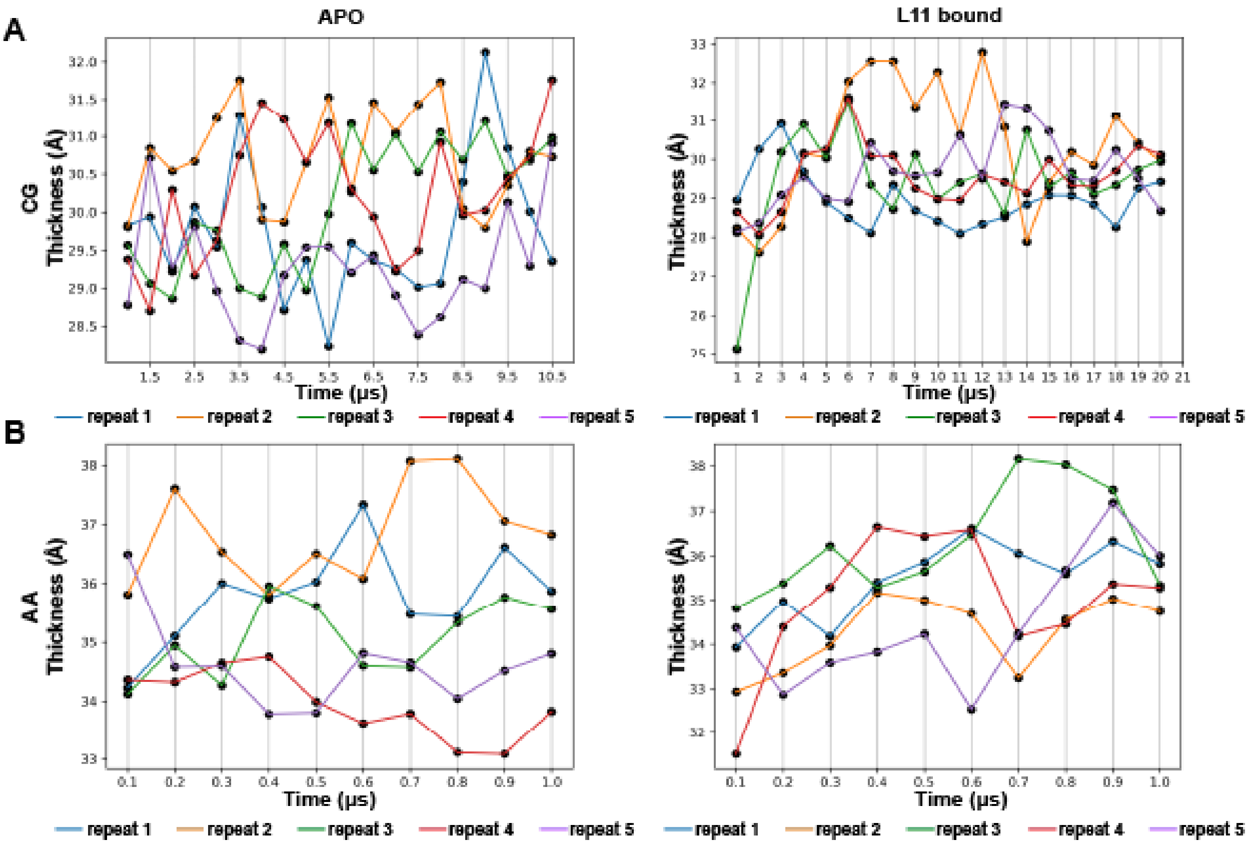

A
APO
L11 bound
CG
Thickness (Å)
Time (μs)
repeat 1
repeat 2
repeat 3
repeat 4
repeat 5
B
AA
Thickness (Å)
Time (μs)
repeat 1
repeat 2
repeat 3
repeat 4
repeat 5

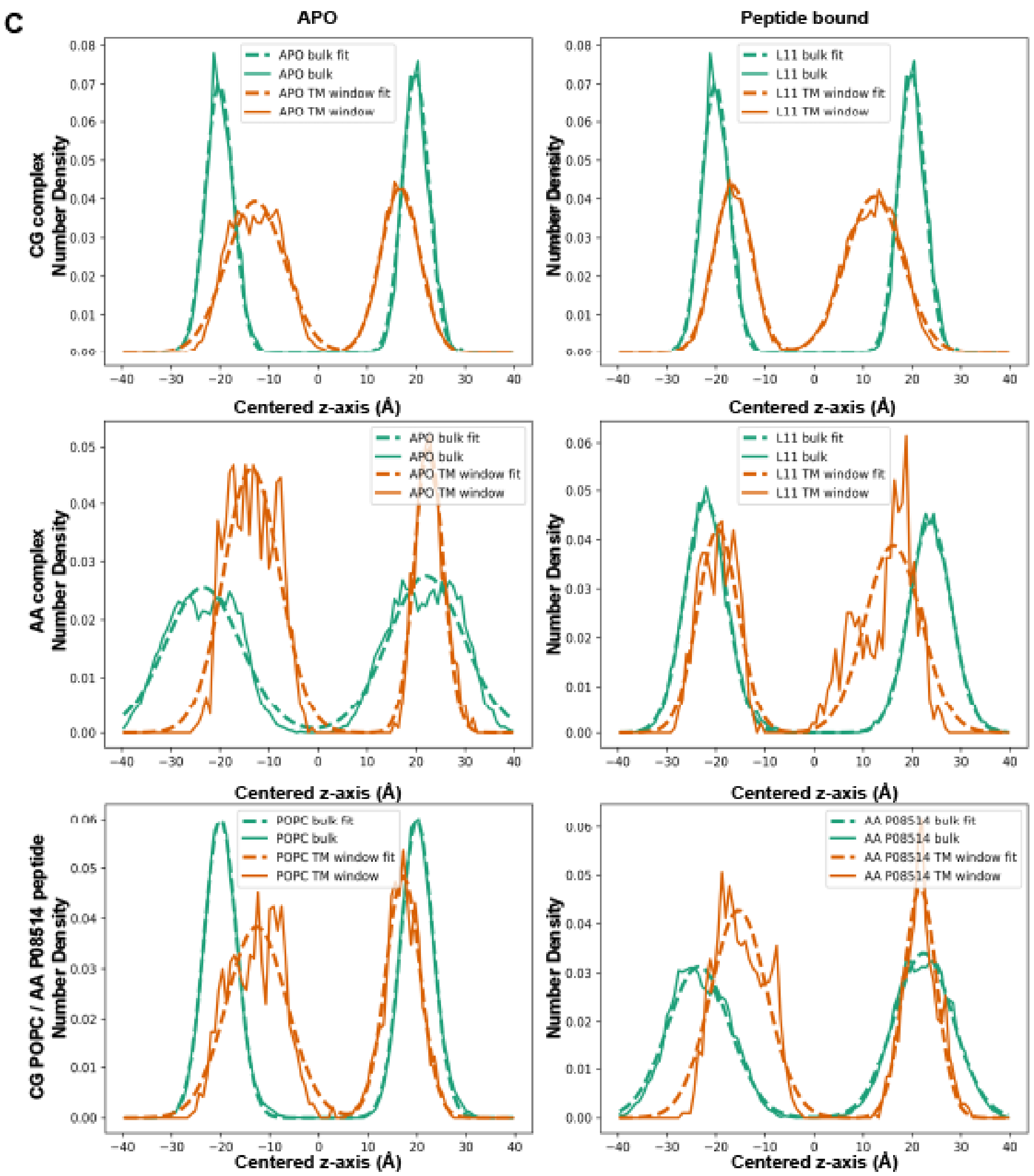

Supplementary Figure 11: Membrane thinning simulations. A-B, Thinning of the membrane in the TM window along simulation time for apo and L11-bound systems at CG and AA resolution, respectively. C, Distribution of lipid head groups found within and outside the TM window in the different systems and resolutions.

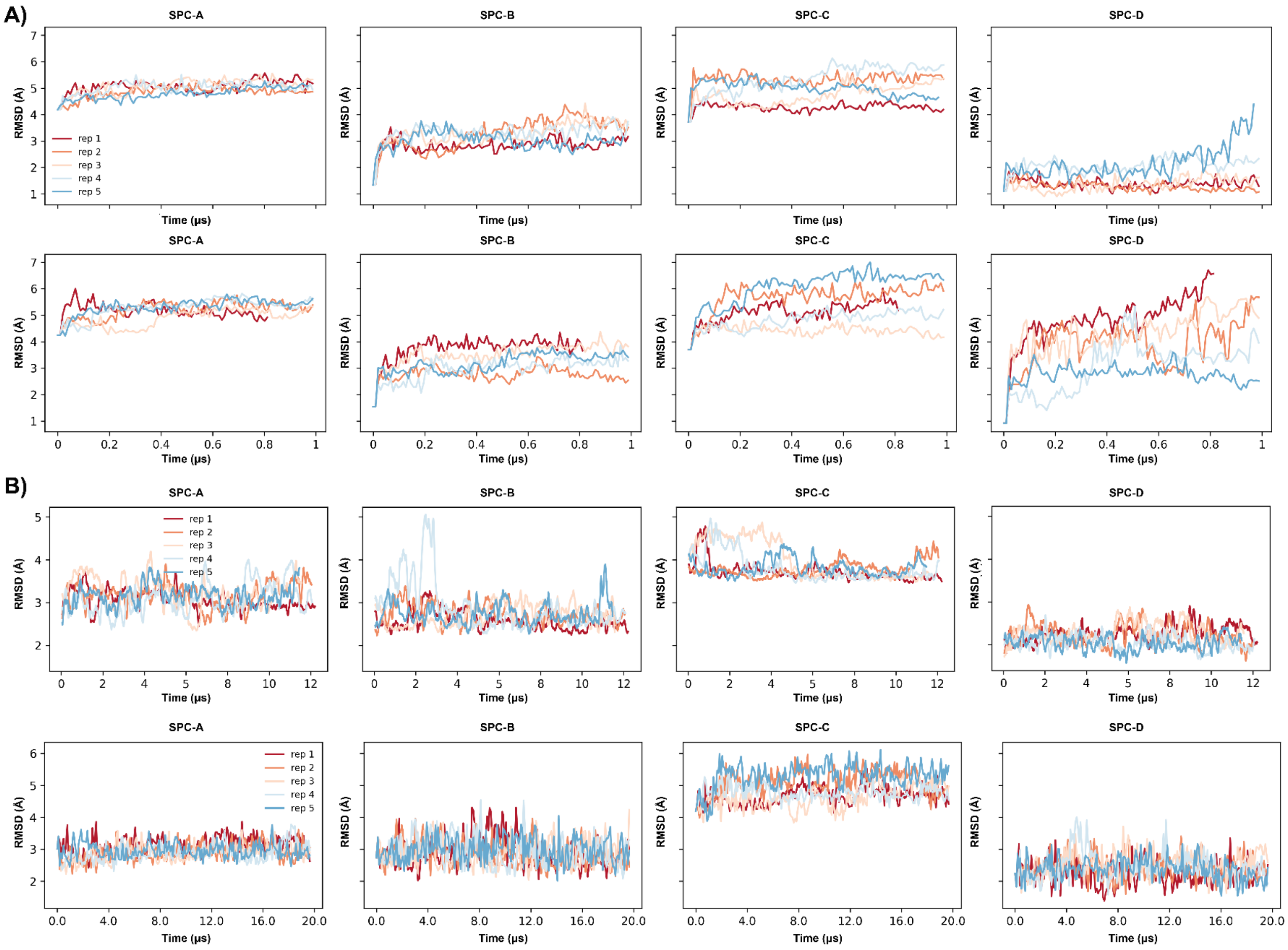
A)
SPC-A
SPC-B
SPC-C
SPC-D
RMSD (Å)
Time (μs)
rep 1
rep 2
rep 3
rep 4
rep 5
B)
SPC-A
SPC-B
SPC-C
SPC-D
RMSD (Å)
Time (μs)
rep 1
rep 2
rep 3
rep 4
rep 5

Supplementary Figure 12: Chain RMSD along MD simulations. A-B, RMSD of the different chains in the protein complex at AA (A) and CG (B) resolution. The complex was first fitted onto SPC-A and then RMSD was obtained for all chains separately.

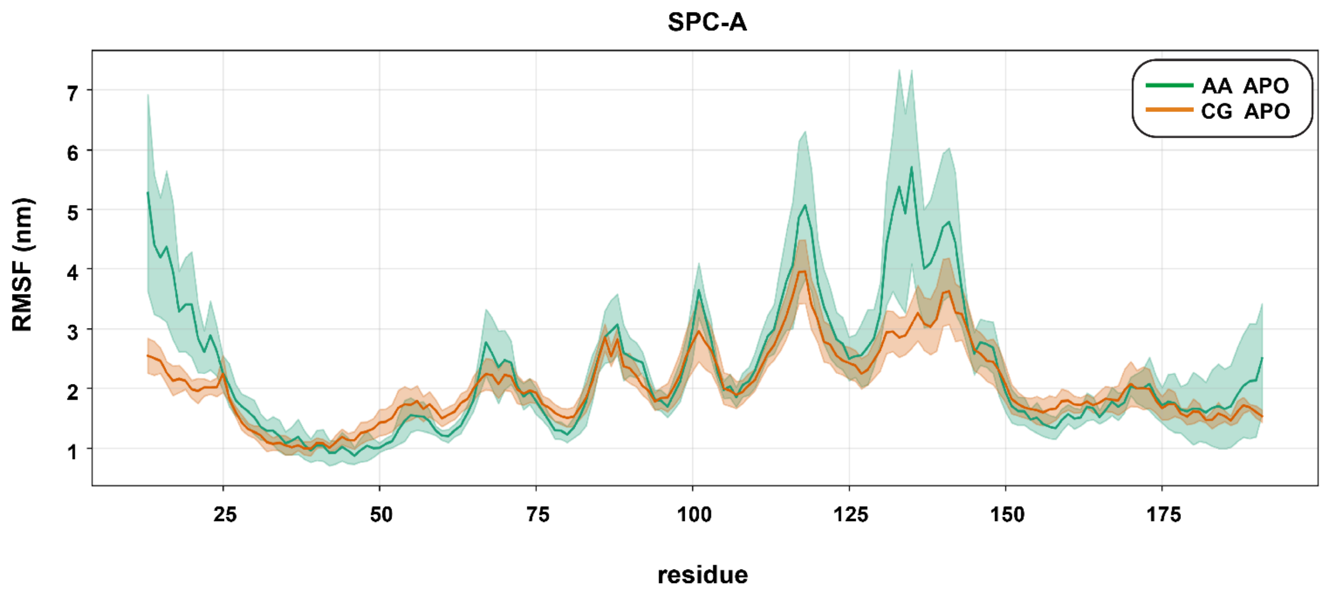

Supplementary Figure 13: RMSF of SPC-A between AA and CG.

Supplementary Tables

Supplementary Table 1: Cryo-EM data collection, refinement and validation statistics

	#1 SPC-$A^{S56A}SP^{L11}$ (EMDB-54010) (PDB 9RJB)	#2 apo SPC-A (EMDB-54011) (PDB 9RJC)
**Data collection and processing**		
Magnification	165,000	130,000
Voltage (kV)	300	300
Electron exposure (e–/Å^2)	60	49,75
Defocus range (μm)	-0.8, -1.0, -1.2	-0.7, -1.0, -1.3, -1.6, -1.9, -2.2, -2.5, -2.8, -3.1,
Pixel size (Å)	0.73	0.73
Symmetry imposed	-	
Initial particle images (no.)	1,743,807	2,475,714
Final particle images (no.)	226,660	516,615
Map resolution (Å)	2.5	4.2
FSC threshold	0.143	0.143
**Refinement**		
Map sharpening *B* factor (Å^2)	57.2	245.3
Model composition		
Non-hydrogen atoms	4,749	4,299
Protein residues	589	534
Ligands	0	0
B factors (Å^2)		
Protein (min/max/mean)	7.91/89.39/45.78	20.89/155.16/61.60
Ligand	-	-
R.m.s. deviations		
Bond lengths (Å)	0.004	0.005
Bond angles (°)	0.567	0.809
Validation		
MolProbity score	1.26	1.47
Clashscore	4.89	8.78
Poor rotamers (%)	0.19	0.21
Ramachandran plot		
Favored (%)	98.25	98.45
Allowed (%)	1.75	1.55
Disallowed (%)	0	0

Supplementary Table 2: Overview of molecular dynamics simulation system parameters.

System	Resolution	Peptide	Protein	Bilayer	Box	Repeats	Time length (μs)
Atomistic systems							
SPC-APO	AA	None	SPC	ER	15x15x15	5	1
SPC-L11	AA	L11	SPC	ER	15x15x15	5	1
SPC-P08514	AA	P08514	SPC	ER	15x15x15	5	1
Coarse Grained Systems							
SPC-APO	CG	None	SPC	ER	15x15x15	5	12
SPC-L11	CG	L11	SPC	ER	15x15x15	5	20
SPC-APO	CG	None	SPC	POPC	15x15x15	1	1
Reference Bilayer Systems							
POPC Bilayer	CG	None	None	POPC	15x15x15	1	1
ER Bilayer	CG	None	None	ER	15x15x15	1	1

Table 2: The membranes are all symmetric composing of either POPC or the Endoplasmic Reticulum (ER) membrane (POPC:POPE:POPS:Cholesterol:PI(3,4)P2 with the ratios 44:26:4:15:11).

Supplementary Table 3: Molecular Dynamics Simulations Checklist.

Reliability and reproducibility checklist for molecular dynamics simulations<br>***All boxes must be marked YES by acceptance unless an N/A option is available**	Yes	N/A	Response<br>(Please state where this information can be found in the text)
**1. Convergence of simulations and analysis**			
1a. Is an evaluation presented in the text to show that the property being measured has equilibrated in the simulations (*e.g.* time-course analysis)?	☒		SI, Supplementary Fig. 7D + 11-12
1b. Then, is it described in the text how simulations are split into equilibration and production runs and how much data were analyzed from production runs?	☒		Main text, page 19
1c. Are there at least 3 simulations per simulation condition with statistical analysis?	☒		Main text page 8 & 19. Five repeats were done.
1d. Is evidence provided in the text that the simulation results presented are independent of initial configuration?	☒		Main text, page 19.<br>Same conformation was used with 5 different initial velocities.
**2. Connection to experiments**			
2a. Are calculations provided that can connect to experiments (*e.g.* loss or gain in function from mutagenesis, binding assays, NMR chemical shifts, J-couplings, SAXS curves, interaction distances or FRET distances, structure factors, diffusion coefficients, bulk modulus and other mechanical properties, *etc.*)?	☒		Yes, Overall stability of SCP structure can be connected the cryo-EM data.<br>SI, Figure S7D + S11-12
**3. Method choice**			
3a. Is it described in the text what force field and water model are used and why?	☒		Main text, page 19. Models are described.
3b. Do simulations contain membranes, membrane proteins, intrinsically disordered proteins, glycans, nucleic acids, polymers, or cryptic ligand binding?	☒	☐	Contains membranes and membrane proteins
If 3b is **YES**, are enhanced sampling methods used?	☐	☒	Response not needed if **N/A**
If enhanced sampling methods are used, are the convergence criteria clearly stated?	☐		
If 3b is **YES**, is it explained in the text why or why not enhanced sampling methods are used?	☐		No, but the simulations were performed using the Martini 3 CG model, which is well known for simulations on this kind of systems, speeding up the sampling.
**4. Code and reproducibility**			
4a. Is a table provided describing the system setup, such as simulation box dimensions, total number of atoms, total number of water molecules, salt concentration, lipid composition (number of molecules and type)?	☒		A table is provided, main text, page 20 (Supplementary Table 2).
4b. Is it described in the text what simulation and analysis software and which versions are used?	☒		Main text, page 18-22

4c. Are initial coordinate and simulation input files and a coordinate file of the final output provided as supplementary files or in a public repository?	☒		Main text, page13-14
4d. Is there custom code or custom force field parameters?	☒	☐	The elastic network added on top of the SPC Martini 3 CG model has some modifications to improve the overall structure and dynamics, as previously shown for these systems (DOI:10.1016/j.molcel.2021.07.031).
If **YES**, are they provided as supplementary profiles or in a public repository?	☒		These a few extra harmonic bonds described in the Main text, page 13-14, and include in the itp files added as part of the SI materials via zenodo link.

Uncropped gels

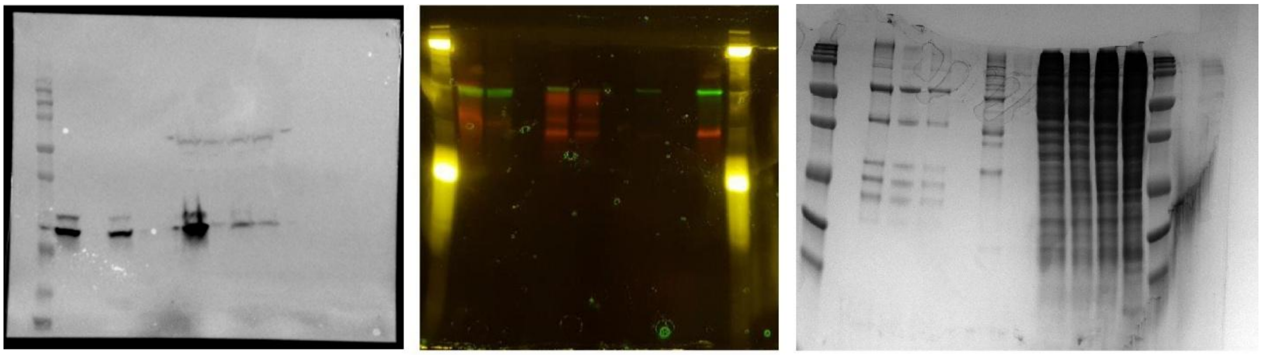

Uncropped gels from Supplementary Figure 4A-B.
