# Supplementary material for: Structural basis of signal peptide recognition by the signal peptidase complex
Source: Nat Commun. 2026 May 22;17:6748. doi: 10.1038/s41467-026-73423-3 (PMC13385932; doi:10.1038/s41467-026-73423-3)
Supplement: Supplementary file 2 — Reporting Summary [file 41467_2026_73423_MOESM2_ESM.pdf]

nature portfolio

Corresponding author(s): Friedrich Förster

Last updated by author(s): Apr 12, 2026

Reporting Summary

Nature Portfolio wishes to improve the reproducibility of the work that we publish. This form provides structure for consistency and transparency in reporting. For further information on Nature Portfolio policies, see our Editorial Policies and the Editorial Policy Checklist.

Statistics

For all statistical analyses, confirm that the following items are present in the figure legend, table legend, main text, or Methods section.

n/a	Confirmed	
☒	☐	The exact sample size (*n*) for each experimental group/condition, given as a discrete number and unit of measurement
☒	☐	A statement on whether measurements were taken from distinct samples or whether the same sample was measured repeatedly
☒	☐	The statistical test(s) used AND whether they are one- or two-sided<br>*Only common tests should be described solely by name; describe more complex techniques in the Methods section.*
☒	☐	A description of all covariates tested
☒	☐	A description of any assumptions or corrections, such as tests of normality and adjustment for multiple comparisons
☒	☐	A full description of the statistical parameters including central tendency (e.g. means) or other basic estimates (e.g. regression coefficient) AND variation (e.g. standard deviation) or associated estimates of uncertainty (e.g. confidence intervals)
☒	☐	For null hypothesis testing, the test statistic (e.g. *F*, *t*, *r*) with confidence intervals, effect sizes, degrees of freedom and *P* value noted<br>*Give P values as exact values whenever suitable.*
☒	☐	For Bayesian analysis, information on the choice of priors and Markov chain Monte Carlo settings
☒	☐	For hierarchical and complex designs, identification of the appropriate level for tests and full reporting of outcomes
☒	☐	Estimates of effect sizes (e.g. Cohen's *d*, Pearson's *r*), indicating how they were calculated

Our web collection on statistics for biologists contains articles on many of the points above.

Software and code

Policy information about availability of computer code

Data collection	Single particle cryo-EM data were acquired using EPU 3.
Data analysis	For cryo-EM single particle analysis we used CryoSPARC 3.3.1 For atomic model building and assessment we used Phenix 1.20.1, Coot 0.9.8.3, Molprobity 4.5.1, and ModelAngelo 1.0.1<br>For modeling of different SPC-SP complexes we used Alphafold3<br>Molecular Dynamics simulations were performed using: PROPKA 3.0, INSANE, Gromacs 2020, AlphaFold2-Multimer (v2), Martinize, CG2AT, CHARMM-GUI, PDB2PQR, MDAnalysis, NumPy, ProLif<br>The code used for structural filtering and positional deviation analysis is available on GitHub at https://github.com/SBC-Utrecht/Sec11A-Signal-Peptide-Analysis. The version of the code used for the final analysis is archived on Zenodo under DOI: 10.5281/zenodo.18620566.<br>Figures were prepared with: UCSF ChimeraX 1.9

For manuscripts utilizing custom algorithms or software that are central to the research but not yet described in published literature, software must be made available to editors and reviewers. We strongly encourage code deposition in a community repository (e.g. GitHub). See the Nature Portfolio guidelines for submitting code & software for further information.

Data

Policy information about availability of data

All manuscripts must include a data availability statement. This statement should provide the following information, where applicable:

- Accession codes, unique identifiers, or web links for publicly available datasets
- A description of any restrictions on data availability
- For clinical datasets or third party data, please ensure that the statement adheres to our policy

Data generated in this study are available in the main article, supplementary materials or in public repositories: nos. EMD-54010, EMD-54011 (www.ebi.ac.uk/emdb) and PDB-9RJB, PDB-9RJC of PDB (www.rcsb.org).

Research involving human participants, their data, or biological material

Policy information about studies with human participants or human data. See also policy information about sex, gender (identity/presentation), and sexual orientation and race, ethnicity and racism.

Reporting on sex and gender	n/a
Reporting on race, ethnicity, or other socially relevant groupings	n/a
Population characteristics	n/a
Recruitment	n/a
Ethics oversight	n/a

Note that full information on the approval of the study protocol must also be provided in the manuscript.

Field-specific reporting

Please select the one below that is the best fit for your research. If you are not sure, read the appropriate sections before making your selection.

[x] Life sciences   [ ] Behavioural & social sciences   [ ] Ecological, evolutionary & environmental sciences

For a reference copy of the document with all sections, see nature.com/documents/nr-reporting-summary-flat.pdf

Life sciences study design

All studies must disclose on these points even when the disclosure is negative.

Sample size	23,425 movie stacks were collected for the apo-SPC dataset; 10,119 movie stacks were collected for the SPC-SPL11 dataset
Data exclusions	After patch CTF and Motion correction, movie stacks with poor CTF and considerable motion were excluded
Replication	n/a
Randomization	n/a
Blinding	n/a

Reporting for specific materials, systems and methods

We require information from authors about some types of materials, experimental systems and methods used in many studies. Here, indicate whether each material, system or method listed is relevant to your study. If you are not sure if a list item applies to your research, read the appropriate section before selecting a response.

Materials & experimental systems

n/a	Involved in the study
☐	☒ Antibodies
☐	☒ Eukaryotic cell lines
☒	☐ Palaeontology and archaeology
☒	☐ Animals and other organisms
☒	☐ Clinical data
☒	☐ Dual use research of concern
☒	☐ Plants

Methods

n/a	Involved in the study
☒	☐ ChIP-seq
☒	☐ Flow cytometry
☒	☐ MRI-based neuroimaging

Antibodies

Antibodies used	Prolactin AntIbody (PA5-79873)
Validation	https://www.thermofisher.com/antibody/product/Prolactin-Antibody-Polyclonal/PA5-79873

Eukaryotic cell lines

Policy information about cell lines and Sex and Gender in Research

Cell line source(s)	Suspension HEK293-E: ImmunoPrecise Antibodies; suspension K562: American Type Culture Collection (CCL-243)
Authentication	Cells were not authenticated
Mycoplasma contamination	Cell lines were not tested for mycoplasma contamination
Commonly misidentified lines (See ICLAC register)	No commonly misidentified cell lines were used in this study

Plants

Seed stocks	n/a
Novel plant genotypes	n/a
Authentication	n/a
