# Supplementary material for: Structural basis of signal peptide recognition by the signal peptidase complex
Source: Nat Commun. 2026 May 22;17:6748. doi: 10.1038/s41467-026-73423-3 (PMC13385932; doi:10.1038/s41467-026-73423-3)
Supplement: Supplementary file 4 — Source Data 1 [file 41467_2026_73423_MOESM4_ESM.pdf]

nature portfolio

Peer Review File

Structural basis of signal peptide recognition by the signal peptidase complex

Corresponding Author: Professor Friedrich Förster

This file contains all reviewer reports in order by version, followed by all author rebuttals in order by version.

Version 0:

Reviewer comments:

Reviewer #1

(Remarks to the Author)
This manuscript by Liaci et al. extends their groundbreaking 2021 work on the human signal peptidase complex (SPC) by refining the apo structure and presenting a substrate-bound cryo-EM model. The study is technically impressive, with high-resolution data (2.6 Å for the enzyme–substrate complex) and careful validation through molecular dynamics simulations. These advances allow the authors to propose mechanistic features, including a transition gate and substrate-binding pockets, and to support their earlier membrane-thinning hypothesis.

While the structural data are of high quality, some of the mechanistic interpretations remain preliminary. In particular, the functional roles of the proposed gatekeeper residues and alternative binding modes suggested by AlphaFold predictions are not yet fully resolved. Additional functional characterization could help to further substantiate these conclusions, although such experiments may be technically challenging.

Overall, this manuscript provides important structural insights into SPC function and represents a significant technical achievement. The work lays a strong foundation for future mechanistic studies of this essential membrane protease.

Reviewer #2

(Remarks to the Author)
Review of manuscript of Liaci et al. entitled: ‘Structural basis of signal peptide recognition by the signal peptidase complex’

This manuscript deals with the important question of how diverse signal peptide sequences are specifically recognized and cleaved by the signal peptidase complex (SPC) after their initial targeting to the Sec61 complex. Since the ER membrane contains a multitude of possible amphipathic substrates, it is important and interesting how SPC can selective distinguish and process hydrophobic signal peptides and not other proteins. Earlier structural work from the authors had revealed the structure of two SPC paralogs in the apo state and provided a structural hypothesis for signal peptide recognition, which this manuscript now attempts to prove through determination of SPC bound to a functional signal peptide. This manuscript reports determination of the SPC-A paralog apo state to higher resolution and the same complex bound to a model SP substrate with a catalytically inactive SPC-A complex. The fact that a co-expressed model SP copurifies with the recombinant SPC-A complex, suggests a stable interaction that survives detergent solubilization and protein purification. The authors demonstrate stable key interactions of the model SP by structural modeling and MD simulations. Finally, the authors used Alphafold3 modeling to examine putative binding poses of variable signal peptide sequences.

This work is an important extension to the insights gained from the earlier apo-SPC structures and provides new understanding into selection and initial stabilization of the signal peptide before cleavage. Remaining questions include how SPC selects among diverse signal peptides and how SPC client polypeptides are transferred from Sec61. The structure with the model signal peptide provides a high resolution view of the interactions of one signal peptide, but given the large diversity in sequences of ER targeting signal peptides, it remains unclear whether the observed binding mode is universal for all signal peptides.

Major points:

1. Given the large sequence diversity of signal peptides, including variation in their lengths and biophysical properties, it remains unclear whether all of them can be recognized at the different levels of ER targeting with same or similar principles. While this manuscript goes a long way into providing the answer for the SPC, I was still left wondering how universal the presented structural example is for diverse signal peptides. In other words, do diverse signal peptides bind SPC in the same or similar stable conformation as the model signal peptide tested in this study? I would request the authors attempt to test further natural signal peptides use MD to analyze their binding to SPC by carrying out additional MD simulations.
2. The model SP is described in the literature, but the authors should also independently demonstrate that this sequence functions as a signal peptide and is correctly processed either in cells or in an in vitro protein translocation system.
3. One question that remains to be discussed and speculated is how the part of the nascent polypeptide C-terminal to the signal peptide might affect binding and processing by SPC. Could the authors discuss what is the typical length of the polypeptide during cleavage and how the distance restraints posed by the C-terminal sequence emanating from the ribosome/Sec61 complex may influence SPC recognition and SP cleavage during cotranslational ER insertion?
4. I wonder if membrane thinning is the predominant way by which the SPC can discriminate between signal peptides and other hydrophobic/amphipatic membrane helices in the ER membrane. Could the authors comment on whether any instances of signal peptides with exceptionally long h-regions have been reported?

Minor points:

1. Abstract. Contains typos, please check spelling. At least sentence “The signal peptidase complex (SPC) is responsible for the cleaving signal peptides”.
2. Page 3. “Signal sequences have the highest specificity at positions P1 and P3 upstream the cleavage site.”. This sentence is somewhat unclear. Do the authors mean that this site is most conserved or defined by presence of specific residues?
3. Page 4. Please provide the actual reference indicated by “ref” currently.
4. Modeling of the SP-engaged state. Thank you for providing the Chimera map and model file, which is ideal for evaluating map and model quality. Could the authors elaborate how the correct registry of the signal peptide was confirmed during model building? In the provided map, it is not immediately clear to me how the sequence registry was confirmed.
5. Page 7. and 16. What was the reason to use a SPC structural model generated by AlphaFold2-Multimer instead of the experimentally determined cryo-EM structure for MD simulations?
6. Page 8. Authors state “The reduction of the thickness of the lipid bilayer compared to areas distal from the SPC is slightly more pronounced when the SP is present”. Can the authors comment on how robust this small difference in bilayer thickness is and support this by for example calculating p-values to represent the robustness of the observed difference.
7. Page 16. Please consider sharing the MD data using a public repository such as Zenodo to enable reproduction of the simulations.
8. Page 17. Use of “newest version of Martinize”, please provide version numbers for the software that was used.
9. Page 17. Can the authors provide motivation for using the specified lipid composition for the MD simulations? Specifically, what was the reason to include this particular amount of cholesterol given the low steady state cholesterol amount in the ER membrane?
10. Page 17. Authors state “Additional harmonic bonds were applied across the monomers to stabilize the complex further”. Please elaborate what these restraints were based on and how they impacted the obtained results.
11. Page 17. Sentence “with a semi-isotropic pressure coupling and a compressibility of bar-1” seems to be missing a value. Please correct.
12. Page 17. Please justify why velocity rescale thermostat was used instead of Nosé–Hoover thermostat that is more common for the CHARMM36 force field.
13. Page 18. Authors state that 5 repeated production runs were carried out, but it is not clear to me how they differ. Please elaborate whether these were truly independent repeats and what was the variability across runs.
14. Page 19. The membrane thickness value was defined as 8 Å, what was the motivation to choose this cutoff value and how many lipids are contained in this region. How sensitive are the obtained results for the chosen cutoff distance?
15. Given that most signal peptides are delivered to SPC cotranslationally once they emerge from Sec61, could the authors speculate about the possible distance of Sec61-SPC handover? I am wondering how proximity of Sec61 might influence membrane thinning around SPC.
16. Page 19. The authors state “Aan initial set of N = 412 SPs [55], was used for SP-modeling.”. Please correct typo in sentence and reference [55] is missing. Also, I wonder why a set of 412 signal peptides was selected? Human proteome contains >3000 signal peptides so I am wondering where the initial set came from and are all from human?

Reviewer #3

(Remarks to the Author)
The manuscript by Liaci et al reports a high-resolution structure of the human signal peptidase complex (SPC-A) bound to a signal peptide. This work represents a substantial advance over the previously published medium-resolution structure and provides important insights into the general binding mode of signal peptides during substrate cleavage. This reviewer has several questions regarding the structural interpretation and data presentation that need be addressed before publication:

1. In the density map of the SPCA-L11 structure, several strong density features are visible at the periphery of the TM regions. These appear significantly stronger than the micelle density. Could the authors clarify what these densities correspond to?

2. In the current model, the authors placed an alternative trace of the substrate peptide into the S1 pocket to account for a density fragment. However, the backbone placement in this region appears somewhat strained and awkward. It would be more convincing to discuss alternative interpretations, such as: a possible U-turn of the substrate after Pro24, density arising from alternative side-chain conformations rather than the main chain, or the possibility of local flexibility/disorder.

3. The labeling of protein bands in Fig. S2D is missing. It is difficult to identify which bands represent the target proteins versus co-purified contaminants. Specifically, the co-purified signal peptide is not labeled. Furthermore, two gels are shown side by side: the left panel is presumably a Coomassie-stained gel, but the nature of the right panel is not specified.

4. The manuscript repeatedly refers to a "β-strand-like" conformation of the signal peptide. Since this feature seems important to the structural analysis, it should be explicitly defined the first time it is introduced (line 130). For example, how does it compare to canonical β-strands (in terms of hydrogen bonding, backbone dihedral angles, or geometry)? Providing a definition will help readers follow later discussions.

5. In the description of the substrate peptide (line 125), residues P7–8, P10–12, and P14–15 are mentioned but not labeled in Fig. 2A. Adding these labels would improve clarity and allow readers to follow the text more easily.

6. The manuscript defines "gatekeeper" residues at line 131. Are these residues conserved across species? If so, an alignment or conservation analysis would strengthen the point. If not, please discuss whether their role is specific to human SPC-A or potentially a more general feature of the complex.

Reviewer #4

(Remarks to the Author)

The manuscript combines structural biology (cryo-EM) with computation of the signal peptide - signal peptide complex (SP - SPC). The 2.6Å cryo-EM structure of the complex will likely become a reference in the field. Computations include classical mechanical MD and AlphaFold structural prediction. A major feature the authors report as having found for the SP-SPC binding is the thinning of the membrane.

The observation of membrane thinning near an intramembrane protease is not new. It was discussed at length for the GlpG protease based on structural biology and MD - see the MD papers from the labs of Stephen H. White, the exp/theory papers from Sinisa Urban's lab, work from the Lemberg lab. The functional role of membrane thinning for substrate binding to the protease has been already discussed for GlpG - see the papers from the White group, and from Sinisa Urban's. These papers need to be cited and the discussion of membrane thinning near the signal protease toned down and presented in the context of these earlier works on GlpG. Likewise, the role of hydrogen-bonding between the protease and the surrounding membrane has already been discussed for GlpG - for which extensive MD and biochemistry data have been published in different lipid membrane environments (Stephen White lab, Urban & Wolf PNAS 2005, see also see also https://doi.org/10.1021/acs.jpcb.8b11291). Those papers need to be cited, and the novelty of the current findings, toned down.

AlphaFold pLDDT scores of 70s are actually at the lower limit of acceptable values for any further consideration of the models. The authors need to clarify whether the relative orientation of the helices is predicted correctly in their dataset of SP sequences, and what is the independent experimental validation used in this part of the work. Likewise, referring to structural models whose plDDT scores are ~65-70 as structures that 'may have only minor inaccuracies' is misleading. That the AlphaFold models 'strikingly converge' may simply be due to the fact that the same templates are used, and to having the same errors in the structural prediction. The library of templates used, and their individual accuracy, need to be discussed before any AlpfaFold model is considered for discussion. Given the pLDDT scores and lack of independent experimental validation, it is rather astonishing that the short Conclusions section even mentions the AlphaFold models.

Protonation states were assigned using PROPKA. While this is a fast method widely used, its limitations for buried protein sites have been discussed at length. The authors need to list all non-standard protonation states, and justify this choice based on experimental data (if available) and clear discussion of the protein electrostatic environment that could shift the pKa value.

It is unclear whether the definition used to monitor the tilt angle of the SP is appropriate for a Pro-containing peptide, as there might be significant internal dynamics of the peptide.

Figures presented in the manuscript are all excellent. However, some clear time series figures showing convergence of parameters computed from MD ought to be included.

Reviewer #5

(Remarks to the Author)
The manuscript entitled "Structural basis of signal peptide recognition by the signal peptidase complex," authored by Förster et al, has a combination of Cryo EM and MD simulation studies. I have a few questions regarding the technical aspect of the

manuscript as follows.

1. Line 71 “as mimicking inhibitors bind to related bacterial signal peptidases (ref) the h-region could be”. No reference is written.

2. A graph corresponding to the change in the membrane head group with the z-axis will be beneficial to understand the membrane thinning effect.

3. How was the tilt angle calculated? What was the vector involved?

4. Justification of the lipid composition taken. Does the lipid composition have something to do with the membrane thinning? If we take a different membrane composition, do we have the same effect?

A lot of studies have already shown that the membrane thinning effect depends on the membrane composition. Therefore, this creates many more questions. A different membrane composition can give a different thinning effect.

5. It is speculated that “The reduction of the thickness of the lipid bilayer compared to areas distal from the SPC 172 is slightly more pronounced when the SP is present (~25%) compared to a simulation performed 173 for the apo SPC-A (~22%). ” Do authors have any particular reason for that? How is the presence of SP helping in the membrane thinning?

Further, there is hardly a difference of 3 %; is it a significant difference to show the impact of SP?

6. What do the authors mean by the “β-sheet style hydrogen bond ”? Do they include certain criteria?

7. What is the location of P1, P5, P2, P4 ? It will be helpful if it is depicted in the graph.

8. The author should clearly mention the drawbacks of the previous Cryo EM structure of SPC A and the novelty of their result in respect to that.

Version 1:

Reviewer comments:

Reviewer #1

(Remarks to the Author)
The authors have addressed all critical points raised during this review.

Reviewer #2

(Remarks to the Author)
The authors have now adequately answered all of my concerns. The manuscript is now ready to be published without further delay.

Reviewer #3

(Remarks to the Author)
The authors have fully addressed my questions, and I recommend the manuscript for publication. It is a very nice piece of work.

Reviewer #4

(Remarks to the Author)
My comments were addressed satisfactorily.

Reviewer #5

(Remarks to the Author)
Most of the comments have been addressed.

Open Access This Peer Review File is licensed under a Creative Commons Attribution 4.0 International License, which permits use, sharing, adaptation, distribution and reproduction in any medium or format, as long as you give appropriate credit to the original author(s) and the source, provide a link to the Creative Commons license, and indicate if changes were made.
In cases where reviewers are anonymous, credit should be given to 'Anonymous Referee' and the source.
The images or other third party material in this Peer Review File are included in the article's Creative Commons license, unless indicated otherwise in a credit line to the material. If material is not included in the article's Creative Commons license and your intended use is not permitted by statutory regulation or exceeds the permitted use, you will need to obtain permission directly from the copyright holder.
To view a copy of this license, visit https://creativecommons.org/licenses/by/4.0/

Rebuttal

We thank the five reviewers for thoroughly reading our manuscript and the constructive suggestions to further improve the manuscript. In particular, we added:

- Validation of SPL11 functioning as a *bona fide* signal peptide using preprolactin as a substrate
- Extended documentation of the molecular dynamics simulations
- Alpha-Fold modeling of all experimentally validated human peptides bound to Sec11A and comparative analysis of high-confidence models

Below we have addressed all points raised and how we revised manuscript accordingly.

Reviewer #1

This manuscript by Liaci et al. extends their groundbreaking 2021 work on the human signal peptidase complex (SPC) by refining the apo structure and presenting a substrate-bound cryo-EM model. The study is technically impressive, with high-resolution data (2.6 Å for the enzyme–substrate complex) and careful validation through molecular dynamics simulations. These advances allow the authors to propose mechanistic features, including a transition gate and substrate-binding pockets, and to support their earlier membrane-thinning hypothesis. We thank Reviewer #1 for appreciating our work.

While the structural data are of high quality, some of the mechanistic interpretations remain preliminary. In particular, the functional roles of the proposed gatekeeper residues and alternative binding modes suggested by AlphaFold predictions are not yet fully resolved. Additional functional characterization could help to further substantiate these conclusions, although such experiments may be technically challenging.

We agree with this assessment. However, as Reviewer #1 points out, such studies are technically very challenging, especially when trying to assess alternative binding modes inside the membrane. Both residues are evolutionary conserved (newly added Figure S5B), and at least gatekeeper residue Trp36 is also likely to be essential for the structural integrity of the SPC.

While we consider such studies beyond the scope of the current manuscript due to the technical challenges involved, we emphasized the need for further experimental studies on page 7.

Overall, this manuscript provides important structural insights into SPC function and represents a significant technical achievement. The work lays a strong foundation for future mechanistic studies of this essential membrane protease.

Thanks.

Reviewer #2

This manuscript deals with the important question of how diverse signal peptide sequences are specifically recognized and cleaved by the signal peptidase complex (SPC) after their initial targeting to the Sec61 complex. Since the ER membrane contains a multitude of possible amphipathic substrates, it is important and interesting how SPC can selective distinguish and process hydrophobic signal peptides and not other proteins. Earlier structural work from the authors had revealed the structure of two SPC paralogs in the apo state and provided a structural hypothesis for signal peptide recognition, which this manuscript now attempts to prove through determination of SPC bound to a functional signal peptide. This manuscript reports determination of the SPC-A paralog apo state to higher resolution and the same complex bound to a model SP substrate with a catalytically inactive SPC-A complex. The fact that a co-expressed model SP copurifies with the recombinant SPC-A complex, suggests a stable interaction that survives detergent solubilization and protein purification. The authors demonstrate stable key interactions of the model SP by structural modeling and MD simulations. Finally, the authors used Alphafold3 modeling to examine putative binding poses of variable signal peptide sequences.

This work is an important extension to the insights gained from the earlier apo-SPC structures and provides new understanding into selection and initial stabilization of the signal peptide before cleavage. Remaining questions include how SPC selects among diverse signal peptides and how SPC client polypeptides are transferred from Sec61. The structure with the model signal peptide provides a high resolution view of the interactions of one signal peptide, but given the large diversity in sequences of ER targeting signal peptides, it remains unclear whether the observed binding mode is universal for all signal peptides.

Major points:

1. *Given the large sequence diversity of signal peptides, including variation in their lengths and biophysical properties, it remains unclear whether all of them can be*

recognized at the different levels of ER targeting with same or similar principles. While this manuscript goes a long way into providing the answer for the SPC, I was still left wondering how universal the presented structural example is for diverse signal peptides. In other words, do diverse signal peptides bind SPC in the same or similar stable conformation as the model signal peptide tested in this study? I would request the authors attempt to test further natural signal peptides use MD to analyze their binding to SPC by carrying out additional MD simulations.

In the revision, we have modeled all experimentally validated human signal peptides and compared the high-confidence models. This analysis shows that all high-confidence models (92 of a 412) closely resemble the experimental structure of SP^{L11} in the c- and h-region (page 10). Thus, the experimental SP^{L11} is likely representative for a large fraction of signal peptides, possibly all of them, which further advances in protein structure prediction might answer.

2. *The model SP is described in the literature, but the authors should also independently demonstrate that this sequence functions as a signal peptide and is correctly processed either in cells or in an in vitro protein translocation system.*

   We used one of the best-characterized secreted protein, (pre)prolactin, to verify that the model SP functions as a *bona fide* signal peptide. Specifically, we show that SP^{L11} fused to preprolactin results in secretion of prolactin from HEK293 cells, comparable to the native preprolactin signal peptide. The added panel Figure S4 A summarizes these results.

3. *One question that remains to be discussed and speculated is how the part of the nascent polypeptide C-terminal to the signal peptide might affect binding and processing by SPC. Could the authors discuss what is the typical length of the polypeptide during cleavage and how the distance restraints posed by the C-terminal sequence emanating from the ribosome/Sec61 complex may influence SPC recognition and SP cleavage during cotranslational ER insertion?*

   We added the section '*SPC structure in context with ER translocon*', also addressing minor point 15:

   In the cellular context, SP cleavage occurs after translocation of the SP towards the ER lumen via the translocon core component Sec61, which is predominantly a co-translational process in mammals. Although *ex vivo* assays showed that SP cleavage can be accomplished post-translationally (Jackson and Blobel 1980), this process is believed to mostly occur co-translationally in the cell (Blobel and Dobberstein 1975). Indeed, *ex vivo* experiments demonstrated that the SPC can function co-

translationally, most comprehensively in (Wollenberg and Simon 2004). These studies indicated that approximately 40 amino acids are required between Sec61 exit and SPC active site for co-translational cleavage. Consistent with the notion of co-translational processing, chemical cross-linking indicated transient proximity of the Sec61 subunit Sec61β and SPC subunit SPCS2 (Kalies, Rapoport et al. 1998). The SPC-SP^{L11} structure indicates that proximal positioning of SPCS2 to Sec61β would position the SPC close to the lateral gate of Sec61 enabling direct insertion of the SP into the SPC membrane window. Interestingly, the SPC would then be in the vicinity of the translocon associated protein complex (TRAP), which modulates the ER lipid membrane shape (Karki, Javanainen et al. 2023). Thus, lipid-protein interactions involving ER translocon and SPC may profoundly impact early biogenesis of secretory pathway proteins. Future experiments in the native ER membrane will be required to address the interplay of ER translocon and SPC.

4. *I wonder if membrane thinning is the predominant way by which the SPC can discriminate between signal peptides and other hydrophobic/amphipatic membrane helices in the ER membrane. Could the authors comment on whether any instances of signal peptides with exceptionally long h-regions have been reported?*
   To our knowledge, this is the first experimental structure of a signal peptide that includes the h-region. Thus, our knowledge of SP structures is currently restricted to computational prediction. Assignment by SignalP suggests that there are essentially no h-regions longer than 15 residues among the probed experimentally verified human SPs, as we have already shown in Liaci et al 2021 (Figure 6C). As we already discussed in this paper, there exist unusually long SPs, which have long n-regions though. In the revised manuscript, we briefly recapitulate our previous SignalP analysis of human SPs on page 9.

Minor points:

1. *Abstract. Contains typos, please check spelling. At least sentence "The signal peptidase complex (SPC) is responsible for the cleaving signal peptides".*
   Fixed.

2. *Page 3. "Signal sequences have the highest specificity at positions P1 and P3 upstream the cleavage site.". This sentence is somewhat unclear. Do the authors mean that this site is most conserved or defined by presence of specific residues?*

We rephrased as: “Signal sequences exhibit the lowest amino acid variation at positions P1 and P3 upstream of the cleavage site, with a strong preference for short, nonpolar amino acids such as alanine”. Thus, it is the latter.

3. *Page 4. Please provide the actual reference indicated by “ref” currently.*

   Done.

4. *Modeling of the SP-engaged state. Thank you for providing the Chimera map and model file, which is ideal for evaluating map and model quality. Could the authors elaborate how the correct registry of the signal peptide was confirmed during model building? In the provided map, it is not immediately clear to me how the sequence registry was confirmed.*

   In anticipation of a lower resolution, we included a Tyrosine at position P2 of SP^{L11} which, with its bulky and aromatic sidechain, serves as a density anchor for the SP registry. Since the density for SP^{L11} is continuous and of high quality throughout the c- and h-region, this anchor residue alone should be sufficient to ensure correct registry. In addition, the high resolution we achieved allows us to identify individual sidechains with high confidence, such as ^{P5}Pro, and we could also clearly discern the residues at the boundaries of the h-region (^{P6}Val6 and ^{P18}Lys). Most importantly, the Q-scores of the fitted SP^{L11} model (added as Figure S4 C iii) are on average >0.7, indicating excellent side chain assignment.

5. *Page 7. and 16. What was the reason to use a SPC structural model generated by AlphaFold2-Multimer instead of the experimentally determined cryo-EM structure for MD simulations?*

   The atomic structure derived from the Cryo-EM map was missing several loops and other segments that were not resolved, which we now explain on page 7. We retrieved the Human SPC amino acid sequence and used AlphaFold2-Multimer (AF) to generate a tentative structure which included all the unresolved parts. To ensure that the AF model was physically reasonable, we compared it to the Cryo-EM structure. We found the AF model reproduced the SPC structure correctly, while being complete, and thus we used it in the MD simulations.

6. *Page 8. Authors state “The reduction of the thickness of the lipid bilayer compared to areas distal from the SPC is slightly more pronounced when the SP is present”. Can the authors comment on how robust this small difference in bilayer thickness is and*

support this by for example calculating p-values to represent the robustness of the observed difference.

We thank the reviewer for pointing out this issue, as there was indeed a mistake in the main text document, where we state that there is a reduction of the thickness of the lipid bilayer compared to areas distal from the SPC is slightly more pronounced when the SP is present. There is only a significant difference across the membrane thicknesses computed between the all-atom and coarse-grained simulations, which we clarified in the revision.

7. *Page 16. Please consider sharing the MD data using a public repository such as Zenodo to enable reproduction of the simulations.*

   The Alphafold2-multimer models generated for the analysis, along with the raw MD trajectory data and additional plots are archived in Zenodo under DOI: 10.5281/zenodo.18299233.

8. *Page 17. Use of "newest version of Martinize", please provide version numbers for the software that was used.*

   We used Martinize2 v015.0, as available in the github repository (https://github.com/marrink-lab/vermouth-martinize) and described at length in its associated publication (https://elifesciences.org/articles/90627), as we specify in the revised methods section.

9. *Page 17. Can the authors provide motivation for using the specified lipid composition for the MD simulations? Specifically, what was the reason to include this particular amount of cholesterol given the low steady state cholesterol amount in the ER membrane?*

   For the setup of our simulations, the membrane composition was set to reproduce simulations of the ER membrane from our previous work (Liaci, Steigenberger et al. 2021). Furthermore, to be sure that the thinning effects observed were not membrane composition-dependent at least at a qualitative level, we also simulated one repeat of SPC apo complex in a pure POPC membrane for 1 μs, from there we calculated the thinning using an 8-Å cutoff. The thinning was calculated using block-average (4 blocs, 250 ns each). The average thinning was 25.3%, with the average thickness in the bulk being 40.1 +/- 0.7 % and in the TM window 30.0 +/- 0.2 %, average and standard deviated across the 4 blocks of the simulation. The thinning still persists even in absence of cholesterol and is comparable to that observed in the ER-like membrane

simulation. Hence, we conclude that the specific concentration of cholesterol in the membrane is not the determining factor for membrane thinning.

10. *Page 17. Authors state "Additional harmonic bonds were applied across the monomers to stabilize the complex further". Please elaborate what these restraints were based on and how they impacted the obtained results.*

    To reproduce the dynamical behavior of the SPC in the all-atom MD simulations at the CG resolution, we added these restraints. They were defined between pairs of monomers, so the complex would keep its quaternary structure. To evaluate whether the restraints provide extra stability to the complex and enable the CG models to reproduce the dynamics of the AA simulations, we provide a RMSF plot comparing the AA and the CG simulations. This plot is added to SI as Figure S13.

11. *Page 17. Sentence "with a semi-isotropic pressure coupling and a compressibility of bar-1" seems to be missing a value. Please correct.*

    The compressibility was set to $3*10^{4}$ bar^{-1}, and the value has been added to the materials and methods section.

12. *Page 17. Please justify why velocity rescale thermostat was used instead of Nosé–Hoover thermostat that is more common for the CHARMM36 force field.*

    While the NH thermostat is commonly used for CHARMM36, and does indeed sample from the canonical ensemble, there are some advantages to using the v-rescale thermostat (Bussi, Donadio et al. 2007). The v-rescale thermostat also robustly samples from the canonical ensemble, it does not exhibit as large temperature fluctuations as the NH thermostat does and it tends to converge faster. One caveat is that due to its stochastic nature, the v-rescale thermostat may slightly perturb the natural dynamics. However, it does usually not have a significant effect on the sampled conformations.

13. *Page 18. Authors state that 5 repeated production runs were carried out, but it is not clear to me how they differ. Please elaborate whether these were truly independent repeats and what was the variability across runs.*

    For each simulation, a random seed was set. While the MD simulations started from the same structure, the random seed ensures that different starting velocities are drawn from the Maxwell-Boltzmann distribution. This translates into each simulation starting from the same structure, but each simulation samples from different regions in phase-space. This has now been explained more clearly in the methods section.

14. *Page 19. The membrane thickness value was defined as 8 Å, what was the motivation to choose this cutoff value and how many lipids are contained in this region. How sensitive are the obtained results for the chosen cutoff distance?*

Thinning - selection of cutoff.

For the apo CG systems we tested three different cutoffs, at 6 Å, 8 Å, and 10 Å, and found the same trend across all three values. By selecting the 8 Å cutoff, whole beads are ensured to be selected while being highly selective to the TM window.

That resulted in:

Cutoff in Å	No. lipid heads	Thinning %
6	12.9 +/- 0.2	26.6 +/- 1.6
8	17.9 +/- 0.3	25.5 +/- 1.4
10	25.5 +/- 0.4	22.8 +/- 1.2

Average and std across repeats. This answer has also been added to the M&M section of the manuscript.

15. *Given that most signal peptides are delivered to SPC cotranslationally once they emerge from Sec61, could the authors speculate about the possible distance of Sec61-SPC handover? I am wondering how proximity of Sec61 might influence membrane thinning around SPC.*

    We refer to major point 3 of reviewer #2.

16. *Page 19. The authors state “Aan initial set of N = 412 SPs [55], was used for SP-modeling.”. Please correct typo in sentence and reference [55] is missing. Also, I wonder why a set of 412 signal peptides was selected? Human proteome contains >3000 signal peptides so I am wondering where the initial set came from and are all from human?*

    See also main point 4. These are all experimentally verified human signal peptides, which we have previously analyzed using SignalP in ref. (Liaci, Steigenberger et al. 2021).

    The typo is fixed.

Reviewer #3

The manuscript by Liaci et al reports a high-resolution structure of the human signal peptidase complex (SPC-A) bound to a signal peptide. This work represents a substantial advance over the previously published medium-resolution structure and provides important insights into the general binding mode of signal peptides during substrate cleavage. This reviewer has several questions regarding the structural interpretation and data presentation that need be addressed before publication:

1. *In the density map of the SPCA-L11 structure, several strong density features are visible at the periphery of the TM regions. These appear significantly stronger than the micelle density. Could the authors clarify what these densities correspond to?*

   Reviewer #3 rightfully points out the existence of such densities, especially around the SEC11-A SPCS3 interface. Specific lipids and detergent molecules often appear in high-resolution cryo-EM maps of membrane proteins – yet they are notoriously difficult to assign with confidence. Hence, we refrain from interpreting further and we referred to these densities in the sentence '' 'In addition to protein density, the map also displays various ordered lipid or detergent molecules in the proximity of the TM window, which we did not analyze further (**Figure S4**)' on page 5.

   In addition, there are some unidentifiable densities occurring around the membrane:cytosol interface of SPCS1 and elsewhere at low contour levels. These most likely originate from the tight masking that was necessary to achieve high resolution using local refinement, as tight masks invariably cut through some of the micelle density. However, these map artifacts disappear with appropriate contouring (>0.16).

2. *In the current model, the authors placed an alternative trace of the substrate peptide into the S1 pocket to account for a density fragment. However, the backbone placement in this region appears somewhat strained and awkward. It would be more convincing to discuss alternative interpretations, such as: a possible U-turn of the substrate after Pro24, density arising from alternative side-chain conformations rather than the main chain, or the possibility of local flexibility/disorder.*

   We thank reviewer #3 for this topic, which we extensively discussed during model building. We have considered various options when generating the model but ultimately concluded that the presented alternative trace provides the most convincing explanation of the data.

   A U-turn of the mature peptide is highly unlikely, as the mature portion of the protein is quite long and contains a folded mCherry domain that is not resolved. A turned-back

substrate would hence be sterically impossible. In addition, local disorder originating from SPC-A appears equally unlikely, as it has not been observed in any of the apo structures we have obtained so far. We assessed the possibility of alternative sidechain positioning as well as the presence of a water molecule, but none of these options produced density fits of comparable quality. While it would be theoretically possible that the side chain of $^{P2'}$Tyr (in the mature peptide) accounts for some of the density, the steric strain and the clearly visible connection to the backbone position P2 render this option highly unlikely.

Lastly, we assessed the mechanistic and evolutionary plausibility of our alternative trace by comparison to related structures. There is one such study (Ting, Harris et al. 2016) presenting crystal structures of the ancestral type I SPase SpsB from *S.aureus* in complex with two physiological substrates (Pep1/2; PDBs 4WVH, 4VWI) and one peptide that was turned into an inhibitor by introducing a $^{P1'}$Pro (Pep3; 4WVJ), analogous to our SP^{L11}. The trace shown as alternative conformation in our manuscript agrees very well with both physiological substrates, while our main trace resembles that of a Pep3.

Our main trace does not enter the cleavage site, presumably due to the presence of $^{P1'}$Pro that was necessary for technical reasons. We quote from (Ting, Harris et al. 2016): “In the Pep3 structure, the P1 methyl group does not protrude as far into the S1 pocket, with an average distance of 4.1 Å between the P1 methyl group and the residues of the S1 pocket, compared with 3.8 Å for Pep1 and Pep2. This is likely to be because the rigid proline pyrrolidine ring at the P1′ position prevents the P1 residue from further approaching the enzyme."

However, it appears that, in contrast to Pep3, SP^{L11} cleavage is not fully inhibited, and the SP can still partially be processed by wt SPC-A, which agrees with our own experience that the P1′ Pro is not sufficient to stabilize SP^{L11} *in vivo*, requiring inclusion of the S56A mutation in SEC11A. Hence, some subpopulation of the peptides appears to fully insert into the S1 site. Along those lines, the two post-cleavage SPs (4 WVH, 4VWI) protrude far into the S1 pocket, analogous to our alternative trace. If they were to be artificially extended, they would produce a similar strain on the peptide bond.

We would like to point out that the ‘awkwardly strained’ bond is the scissile bond, and that this steric strain might be a part of the catalytic mechanism. In our cleavage-incompetent construct, this strain cannot be alleviated by a nucleophilic attack and the subsequent stabilization of a tetrahedral intermediate state by the oxyanion hole. However, we refrain from this claim in the manuscript because our densities are not clear enough to provide structural proof and we do not provide detailed kinetic analyses of the cleavage process.

We have added a respective short passage to the main manuscript (page 7).

3. *The labeling of protein bands in Fig. S2D is missing. It is difficult to identify which bands represent the target proteins versus co-purified contaminants. Specifically, the co-purified signal peptide is not labeled. Furthermore, two gels are shown side by side: the left panel is presumably a Coomassie-stained gel, but the nature of the right panel is not specified.*

   The gels only illustrated the tandem affinity isolation. We decided to replace the gel by a gel of the purified sample after size exclusion chromatography, which makes band identification straightforward (Figure S4 B). The bands have been assigned.

   The gel on the right hand side is a blue/green LED gel that images the GFP-tag on SEC11A as well as the mCherry tag on the SP. The figure caption has been modified accordingly.

4. *The manuscript repeatedly refers to a "β-strand-like" conformation of the signal peptide. Since this feature seems important to the structural analysis, it should be explicitly defined the first time it is introduced (line 130). For example, how does it compare to canonical β-strands (in terms of hydrogen bonding, backbone dihedral angles, or geometry)? Providing a definition will help readers follow later discussions.*

   Most commonly, automatic assignment of secondary structure elements is performed by the tool Define Secondary Structure of Proteins (DSSP) by Kabsch and Sander. The standard parameters of this algorithm would not assign the c-region of the SP as a β-strand, yet lowering the H-bond energy cutoff to -0.18 kcal/mol (default in ChimeraX: -0.5 kcal/mol). Since the precise choice of cutoffs is ultimately arbitrary we refer to a β-strand in the revised manuscript and clarify the used parameters for rendering (Figure S5A-i).

5. *In the description of the substrate peptide (line 125), residues P7–8, P10–12, and P14–15 are mentioned but not labeled in Fig. 2A. Adding these labels would improve clarity and allow readers to follow the text more easily.*

   We labeled the SPL11 residues in Figure 2 and also provide an overview of all residues in Figure S4C.

6. *The manuscript defines "gatekeeper" residues at line 131. Are these residues conserved across species? If so, an alignment or conservation analysis would*

strengthen the point. If not, please discuss whether their role is specific to human SPC-A or potentially a more general feature of the complex.

We thank reviewer #3 for this suggestion. We aligned Sec11A from 20 common organisms that cover major parts of eukaryotes, which indeed clearly shows the conservation of the gatekeeper residues (Figure S5B).

Using a diverse set of 20 different organisms, including mammals, amphibians, insects, plants, fungi, and protozoa, after sequence alignment of the Sec11A subunit and conservation analysis, we observe that both gatekeeper residues are perfectly conserved and reside in a residue stretch of high conservation score. Even in the case of *S. pombe* where Trp 36 has been replaced with a Tyr, we still observe the presence of a bulky, polar residue, that can serve a similar function.

Reviewer #4:

The manuscript combines structural biology (cryo-EM) with computation of the signal peptide - signal peptide complex (SP - SPC). The 2.6Å cryo-EM structure of the complex will likely become a reference in the field. Computations include classical mechanical MD and AlphaFold structural prediction. A major feature the authors report as having found for the SP-SPC binding is the thinning of the membrane.

The observation of membrane thinning near an intramembrane protease is not new. It was discussed at length for the GlpG protease based on structural biology and MD - see the MD papers from the labs of Stephen H. White, the exp/theory papers from Sinisa Urban's lab, work from the Lemberg lab. The functional role of membrane thinning for substrate binding to the protease has been already discussed for GlpG - see the papers from the White group, and from Sinisa Urban's. These papers need to be cited and the discussion of membrane thinning near the signal protease toned down and presented in the context of these earlier works on GlpG. Likewise, the role of hydrogen-bonding between the protease and the surrounding membrane has already been discussed for GlpG - for which extensive MD and biochemistry data have been published in different lipid membrane environments (Stephen White lab, Urban & Wolf PNAS 2005, see also see also https://doi.org/10.1021/acs.jpcb.8b11291). Those papers need to be cited, and the novelty of the current findings, toned down.

While GlpG is neither functionally nor structurally/mechanistically related to the SPC, we agree that it makes sense to mention GlpG as another example where protein-lipid interactions impact proteolysis. Novelty has not been claimed in the manuscript.
We refer to the original GlpG works (Urban and Wolfe 2005, Bondar, del Val et al. 2009, Engberg, Ulbricht et al. 2022) in the discussion of the thinning results in the revised manuscript.
"Local membrane thinning has previously been demonstrated to enhance proteolytic activity in the rhomboid protease GlpG, which locally modulates the lipid bilayer around its intramembrane active site (Urban and Wolfe 2005, Bondar, del Val et al. 2009, Engberg, Ulbricht et al. 2022). In contrast to GlpG, the SPC active site resides in the ER lumen. The lipid thinning observed within the SPC transmembrane window in our MD simulations may influence proteolytic function over a considerably greater distance due to the longer recognition motif that characterizes an SP."

AlphaFold pLDDT scores of 70s are actually at the lower limit of acceptable values for any further consideration of the models. The authors need to clarify whether the relative orientation of the helices is predicted correctly in their dataset of SP sequences, and what is the independent experimental validation used in this part of the work. Likewise, referring to structural models whose plDDT scores are ~65-70 as structures that 'may have only minor inaccuracies' is misleading. That the AlphaFold models 'strikingly converge' may simply be due to the fact that the same templates are used, and to having the same errors in the structural prediction. The library of templates used, and their individual accuracy, need to be discussed before any AlpfaFold model is considered for discussion. Given the pLDDT scores and lack of independent experimental validation, it is rather astonishing that the short Conclusions section even mentions the AlphaFold models.
We revised the modeling aspect, making the point of the modeling more explicit, which is effectively to answer point 1 of reviewer #2: what can we expect for the binding modes of the large variety of native signal peptides? To answer this question, we switched to AF2 multimer, which does not restrict us to the AF3 server allowing us to model all 412 experimentally verified human signal peptides. We discarded all models with pLDDT scores <70 and not meeting fundamental properties of signal peptides (topology, cleavage site near catalytic center), resulting in 92 high-confidence models. We validate the AF2 modeling on our experimental SPCA-SP^{L11} structure, which shows strong correlation of pLDDT score with model accuracy. The bottom line of the modeling is that the SPCA-SP^{L11} structure is likely representative for the binding of

many native SPs, and there are no indications of strongly differing modes of interaction.

To maximize accuracy templates were used for modeling, yet these exclusively cover Sec11A. Hence, bias of the SP prediction and respective pLDDT scores is not to be expected. Consistent with this expectation, switching off the use of templates for selected examples did not result in major structural differences.

The conclusion section was shortened, reflecting the main message.

Protonation states were assigned using PROPKA. While this is a fast method widely used, its limitations for buried protein sites have been discussed at length. The authors need to list all non-standard protonation states, and justify this choice based on experimental data (if available) and clear discussion of the protein electrostatic environment that could shift the pKa value.

17. The protonation state of the titratable residues in the reference structure of the SPC tetramer was evaluated using PROPKA3.0, implemented in the APBS webserver. We agree with the reviewer that while widely used, this method has limitations which were discussed at length. However, the results from the PROPKA run we carried did not suggest the need to assign any residues to a non-standard protonation state. Protonation assignments were additionally verified by direct visual inspection to avoid common errors, such as charged residues in hydrophobic transmembrane regions. The PQR file extracted from the reference structure following APBS has been archived in Zenodo under DOI: 10.5281/zenodo.18299233.archived in Zenodo under DOI: 10.5281/zenodo.18299233.

It is unclear whether the definition used to monitor the tilt angle of the SP is appropriate for a Pro-containing peptide, as there might be significant internal dynamics of the peptide.

While there is a Pro at the end of the c-region, the h-region and the corresponding helix do not contain a Pro (the corresponding region is a poly leucine). Thus, we argue that the tilt angle is well defined.

Figures presented in the manuscript are all excellent. However, some clear time series figures showing convergence of parameters computed from MD ought to be included.

18. To address the reviewers' concern, we have added RMSD and tilt angle and thinning of the membrane plots across simulation time (Figure S11, Zenodo DOI: 10.5281/zenodo.18299233). This data, along with the RMSF plot Figure S13 (Reviewer #2, minor point #10), illustrates that the CG simulations carried out were

long enough to converge and that the dynamical properties queries are robust and statistically significant.

Reviewer #5

The manuscript entitled "Structural basis of signal peptide recognition by the signal peptidase complex," authored by Förster et al, has a combination of Cryo EM and MD simulation studies. I have a few questions regarding the technical aspect of the manuscript as follows.

1. *Line 71 "as mimicking inhibitors bind to related bacterial signal peptidases (ref) the h-region could be". No reference is written.*
   Done. See also minor point of reviewer #2.

2. *A graph corresponding to the change in the membrane head group with the z-axis will be beneficial to understand the membrane thinning effect.*
   We thank the reviewer for the comment and have added Figure S11C showing this.

3. *How was the tilt angle calculated? What was the vector involved?*
   We have clarified the methodology for the tilt angle calculation in the revised Methods and Materials section. To define the principal axis of the signal peptide helix, we used a vector-based approach: The helix vector was defined by the displacement between two points: the center of mass of the first three residues and the center of mass of the last three residues of the transmembrane helix. The membrane normal was defined as the z-axis of the simulation box. The tilt angle was calculated as the angle between this helix vector and the membrane normal. The calculation was performed for every frame of the MD trajectory using the MDAnalysis and NumPy libraries. The reported values represent the average angle over the stable portion of the trajectory.
   Justification of the lipid composition taken. Does the lipid composition have something to do with the membrane thinning? If we take a different membrane composition, do we have the same effect? A lot of studies have already shown that the membrane thinning effect depends on the membrane composition. Therefore, this creates many more questions. A different membrane composition can give a different thinning effect.
   As answered to reviewer #2, we also tested a pure POPC bilayer for baseline comparison and found a thinning of 25.3%, not significantly different from the complex membrane composition used. While a different composition can potentially lead to a

different membrane thinning percentage, our data indicates that the trend is robust. This result is from a single simulation of the apo system for 20 μs. We have added Figure S11A-B showing this as well.

4. *It is speculated that "The reduction of the thickness of the lipid bilayer compared to areas distal from the SPC is slightly more pronounced when the SP is present (~25%) compared to a simulation performed for the apo SPC-A (~22%). " Do authors have any particular reason for that? How is the presence of SP helping in the membrane thinning?*

   Further, there is hardly a difference of 3 %; is it a significant difference to show the impact of SP?

   We refer to reviewer #2, minor point 6. We apologize for the error in the Main Text. The corresponding Figure is correct and shows that there is no significant difference between the simulations with and without the peptide bound. However, we do see that there is a difference between the AA and CG simulations, which is likely because of the increased sampling in the CG simulations.

5. *What do the authors mean by the "β-sheet style hydrogen bond"? Do they include certain criteria?*

   See also response to reviewer #3, point 5. We now refer to β-sheet as assignment is dependent on the precise choice of parameters that we specified.

6. *What is the location of P1, P5, P2, P4? It will be helpful if it is depicted in the graph.*

   As also suggested by referee #3, we added labels to Figure 2 and provided a complete overview in Figure S4C.

7. *The author should clearly mention the drawbacks of the previous Cryo EM structure of SPC A and the novelty of their result in respect to that.*

   We added the following into the text: "The improved resolution also allows for a more reliable model building compared to the previous structure, as indicated by substantially higher Q-scores. Notably, this resulted in more accurate tracing of the loop containing the catalytic Asp residue and loops in the cytosolic face of SPC-A."

   Furthermore, we added supplementary panel Fig. S2D, which illustrates the improvement in model quality and the differences in tracing.

References

Blobel, G. and B. Dobberstein (1975). "Transfer of proteins across membranes. I. Presence of proteolytically processed and unprocessed nascent immunoglobulin light chains on membrane-bound ribosomes of murine myeloma." J Cell Biol **67**(3): 835–851.

Bondar, A. N., C. del Val and S. H. White (2009). "Rhomboid protease dynamics and lipid interactions." Structure **17**(3): 395–405.

Bussi, G., D. Donadio and M. Parrinello (2007). "Canonical sampling through velocity rescaling." The Journal of Chemical Physics **126**(1): 014101.

Engberg, O., D. Ulbricht, V. Dobel, V. Siebert, C. Frie, A. Penk, M. K. Lemberg and D. Huster (2022). "Rhomboid-catalyzed intramembrane proteolysis requires hydrophobic matching with the surrounding lipid bilayer." Sci Adv **8**(38): eabq8303.

Jackson, R. C. and G. Blobel (1980). "Post-translational processing of full-length presecretory proteins with canine pancreatic signal peptidase." Ann N Y Acad Sci **343**: 391–404.

Kalies, K. U., T. A. Rapoport and E. Hartmann (1998). "The beta subunit of the Sec61 complex facilitates cotranslational protein transport and interacts with the signal peptidase during translocation." J Cell Biol **141**(4): 887–894.

Karki, S., M. Javanainen, S. Rehan, D. Tranter, J. Kellosalo, J. T. Huiskonen, L. Happonen and V. Paavilainen (2023). "Molecular view of ER membrane remodeling by the Sec61/TRAP translocon." EMBO Rep **24**(12): e57910.

Liaci, A. M., B. Steigenberger, P. C. Telles de Souza, S. Tamara, M. Grollers-Mulderij, P. Ogrissek, S. J. Marrink, R. A. Scheltema and F. Förster (2021). "Structure of the human signal peptidase complex reveals the determinants for signal peptide cleavage." Mol Cell **81**(19): 3934–3948.

Ting, Y. T., P. W. Harris, G. Batot, M. A. Brimble, E. N. Baker and P. G. Young (2016). "Peptide binding to a bacterial signal peptidase visualized by peptide tethering and carrier-driven crystallization." IUCrJ **3**(Pt 1): 10–19.

Urban, S. and M. S. Wolfe (2005). "Reconstitution of intramembrane proteolysis in vitro reveals that pure rhomboid is sufficient for catalysis and specificity." Proc Natl Acad Sci U S A **102**(6): 1883–1888.

Wollenberg, M. S. and S. M. Simon (2004). "Signal sequence cleavage of peptidyl-tRNA prior to release from the ribosome and translocon." J Biol Chem **279**(24): 24919–24922.
